# Drivers of fungal and bacterial communities in ectomycorrhizospheres of birch, oak, and pine in a former uranium mining site, Ronneburg, Germany

**DOI:** 10.1007/s11356-025-36330-6

**Published:** 2025-04-02

**Authors:** Olga Bogdanova, Katrin Krause, Sebastian Pietschmann, Erika Kothe

**Affiliations:** Institute of Microbiology, Microbial Communication, Friedrich Schiller University Jena, Neugasse 25, 07743 Jena, Germany

**Keywords:** Ectomycorrhiza, Community, Metal influence, Plant, Field study, Pot experiment

## Abstract

The impact of soil and tree species on fungal and bacterial communities was investigated in a former uranium mining area with field and pot studies of the mycorrhizospheres of birch (*Betula pendula*), oak (*Quercus robur*), and pine (*Pinus sylvestris*). At the initial stages of succession re-created in the pot experiment, tree-species-specific microbial communities were detected. The pot microbiomes showed lower diversity and evenness of fungi and bacteria as compared to field-grown trees. In the natural field setting, the fungal community both in bulk and rhizosphere soil consisted of mainly *Thelephoraceae*, *Inocybaceae* and *Russulaceae.* They contributed with *Leotiaceae* and *Herpotrichiellaceae* to 52–85% of overall abundances, showing the soil hyphae impact of ectomycorrhiza in the tree stand. The fungal communities and their distribution patterns reflected host tree specificity and successional stage of the ectomycorrhizosphere. In the bacterial community, the most abundant bacterial classes were *Alphaproteobacteria*, *Acidobacteria*, *Ktedonobacteria*, *Bacteroidia*, *Gammaproteobacteria*, and *Phycisphaerae* representing about 59–80% of all bacterial sequences. The bacterial communities correlated with soil chemical parameters, particularly the content of toxic metals, total nitrogen and C/N ratio. This study allowed to identify drivers for microbial community composition, which might be helpful to develop afforestation strategies in post-mining landscapes.

**Supplementary Information:**

The online version contains supplementary material available at 10.1007/s11356-025-36330-6.

## Introduction

The former uranium mining site near Ronneburg (Germany) shows severe ecosystem disturbances and heavy metal contamination. The remediation activities started in 1990 and included re-forestation on the barren soil cover resulting in a primary succession (Iordache et al. 2009; Bogdanova et al. 2023). During primary succession, microorganisms play a central role in soil formation and soil development, initiating C and N cycling and promoting plant growth (Schulz et al. 2013; Ciccazzo et al. 2016). Vegetation modifies soil porosity, aeration, and hydraulic conductivity, and creates specific habitats for microorganisms (de la Fuente Cantó et al. 2020). Root exudates and plant litter contribute significantly to organic matter accumulation in early stages of succession. The quantity and chemical composition of the released compounds are determined by the plant species, stage of plant development as well as soil conditions (Dennis et al. 2010). By coating the soil particles and binding them together, root exudates contribute to the formation of water stable aggregates influencing water content and water holding capacity (Carminati et al. 2017). The exudation of organic acids can change soil pH and redox potential, which affects mobilization and availability of nutrients as well as chelate effectively metal cations, such as Al^3+^ and Fe^3+/2+^, decreasing metal toxicity (de la Fuente Cantó et al. 2020; Osmolovskaya et al. 2018).

These changes, in turn, will have a considerable effect on soil microorganisms, the “rhizosphere effect,” establishing a specific microbial community around the root system, with increased activity and reduced diversity compared to the bulk soil (de la Fuente Cantó et al. 2020). The rhizosphere community includes bacteria, fungi, oomycetes, archaea, algae, viruses, and microfauna (Mendes et al. 2013), some of which form symbiotic interactions with plant roots like mycorrhiza, and selection of a particular rhizobiome is beneficial for plant growth and health (Mendes et al. 2013).

A similar selection process was shown for mycorrhizal fungi which led to the idea of a specific mycorrhizosphere habitat where organisms are dependent on the organic compounds produced by both plant and fungal symbionts (Linderman 1987; Garbaye 1991). Specific bacterial genera, including *Pseudomonas*, *Burkholderia*, and *Bacillus,* have been reported for ectomycorrhizosphere communities and contribute to the effects (Uroz et al. 2016). Many specialized mycorrhizosphere bacteria have been shown to promote plant growth and protect plants against pathogens (plant growth-promoting bacteria, PGPB). Mycorrhiza helper bacteria specifically increase mycorrhization rates (Frey-Klett et al. 2007) and include Proteobacteria (*Agrobacterium*, *Azospirillum*, *Azotobacter*, *Burkholderia*, *Bradyrhizobium*, *Enterobacter*, *Pseudomonas*, *Klebsiella*, *Rhizobium* etc.), Firmicutes (e.g., *Bacillus*, *Brevibacillus*, *Paenibacillus*) and Actinobacteria (*Rhodococcus*, *Streptomyces*, *Arthrobacter*) (Duponnois and Garbaye 1991).

Post-mining substrates are commonly characterized by high concentrations of toxic metals. Bacteria and fungi found in metal-contaminated soils are adapted to metal contamination (Schmidt et al. 2009; Brangsch et al. 2022; Harpke and Kothe 2023) which, in turn, affect mobility and bioavailability of metals in soil. Ectomycorrhizal communities in contaminated soils are usually characterized by a low diversity of morphotypes with a preference for contact and short-distance exploration strategies and mainly dominated by *Russulaceae, Inocybaceae, Cortinariaceae, Thelephoraceae, Rhizopogonaceae, Tricholomataceae*, as well as abundant *Meliniomyces bicolor*. The contact morphotypes correlated with high Al, Cu, Fe, Sr, and U soil contents, the dark-colored short-distance exploration type did not show a specific preference for soil characteristics, and the medium fringe type with rhizomorphs on oaks correlated with the content of total nitrogen (Bogdanova et al. 2023). Studies on microbial community structure in post-mining areas focus on succession, while the effects on remediation schemes are less well researched. Mainly, a so-called “toxicity index” has been applied to explain ectomycorrhizal communities in contaminated soils (Bierza et al. 2020). Here, *Scleroderma*, *Russula*, and *Cortinarius* species were found to correlate with high heavy metal loads. Furthermore, it has been shown that the content of several toxic metals affected the structure of microbial community which resulted in the decrease of *Actinobacteria* and *Acidobacteria* and the increase of relative abundance of *Chloroflexi* and *Ktedonobacteria*, and *Alphaproteobacteria* and *Chlorobi* had a strong positive correlation with pH value (Epelde et al. 2015). A low pH typical for post-mining landscapes leads to higher metal mobility in soil water with a higher bioavailability of (heavy) metals that might influence microbial communities.

The ectomycorrhizosphere in post-mining environments is a typical scenario of primary succession. To separate experimentally the effects of soil, plant, and mycorrhizal symbionts on creating the ectomycorrhizosphere, more standardized conditions are needed. To this end, pot experiments are better suited where a reduced diversity and a shift towards long-distance exploration types have been observed as compared to field studies. Still, the approach of hyper-inoculation mimics a secondary succession in soils where more ectomycorrhizal spores would be present (Bogdanova et al. 2023).

This study aimed to investigate the bacterial and fungal ectomycorrhizosphere communities to identify factors driving microbial community composition with special emphasis on the influence of bioavailable metals. Here, we test the hypotheses of (1) the rhizosphere shaping a specific mycorrhizosphere community, (2) the fungal community in the field depends on vegetation, (3) abiotic conditions primarily influence the bacterial community, and (4) pot experiments can mimic secondary succession. The analysis will allow to get hints in developing afforestation strategies in post-mining landscapes. (5) to get hints in developing afforestation strategies in post-mining landscapes.

## Material and methods

### Soil and plant sampling

Soil and saplings were sampled at Kanigsberg near Ronneburg in the former uranium mining area, Germany (see suppl. Figure S1). The former heap site is characterized by low pH, associated with high loads of bioavailable (heavy) metals. Chemical parameters, e.g. soil pH, total carbon (TC), nitrogen (TN), and phosphorus (TP) contents, and content of bioavailable metals were analyzed from air-dried and sieved soil, see Bogdanova et al. (2023). The three test field sampling sites with birch, oak, and pine showed distinct separation based on differences in soil chemical parameters (suppl. Tables S1-S2). Birch sampling site illustrated a particularly early stage of primary succession with immature soil and total absence of litter and organic horizons. The soil contained a very low amount of TC, TN, TP, and high concentrations of Al, Fe, and Cu compared to other sites. The soil at the oaks area contained a relatively high amount of TC, TN, and TP and increased concentration of Mn. The pine sampling site took a rather intermediate position between the birch and oak sites and shared common abiotic characteristics. Soil chemical analysis showed higher pH values than other sites and exceptionally high variability between soil samples of this sampling site.

Field plant mycorrhizosphere (MR) and soil with distance to tree roots (bulk soil) were carefully taken after removal of the topsoil layer. Sampling was performed for three trees of each species, which were treated further as three individual samples (replicates). Samples were taken from stands of 2–3 years old birch (*Betula pendula* of 25–30 cm*;* 50°49′35.35″ N, 12°9′11.68″ E), oak (*Quercus robur* 7–10 cm high; 50°49′36.13″ N, 12°9′12.56″ E), and pine *(Pinus sylvestris* of 7–11 cm; 50°49′39.65″ N, 12°9′17.87″ E; see suppl. Figure S1). Small trees of approx. 2–3 years were sampled for the pot experiment at the same sites. After removing the topsoil layer, roots with attached soil aggregates (rhizosphere soil) as well as soil not directly connected to tree roots (bulk soil) were taken for comparison (three replicates per tree species).

For the pot experiment, soil was sampled (control pot substrate: sampling site 50°49′35.27″ N, 12°9′12.13″ E; see suppl. Figure S1, (Bogdanova et al. 2023)), pre-sieved, dried for 3 days, sieved (< 2.8 mm), and filled into Mitscherlich pots (12 L with 6 kg of substrate). To prevent direct contact with the pot metal walls, pots were inlaid with polyethylene film. Soil attached to the roots was carefully removed before planting two trees of one species per pot. The pots were incubated in a greenhouse to control abiotic conditions. Pots were watered with deionized water to approximately 15 volume % water content weekly during autumn–winter and twice per week in the spring–summer seasons. To improve air exchange, the topsoil crust was carefully broken with non-metal equipment the day after watering. At least three replicates of pots for each tree species were analyzed after two years (Bogdanova et al. 2023).

### Community sequencing

Soil from the test field or pots was kept in sterile Falcon tubes and processed immediately after sampling. Total soil genomic DNA was extracted from 0.25 g of soil sample using PowerSoil DNA Isolation kit (MoBio, Carlsbad, USA) according to manufacturer’s instructions in triplicates. The concentration of extracted DNA was measured with DeNovix DS-11 Spectrophotometer (Biozym, Hessisch Oldendorf, Germany). Since the yield of DNA was very low, no additional DNA purification step was performed. Triplicates of each sample were pooled and sequenced using the Illumina MiSeq platform at StarSEQ (Mainz, Germany) after amplification of 16S rDNA region (bacteria) using primers 27F (5’—AGA GTT TGA TCC TGG CTC AG—3’) and 534R (5’—ATT ACC GCG GCT GCT GG—3’), and ITS1 region (fungi) using primers ITS1F (5’—CTT GGT CAT TTA GAG GAA GTA A—3’) and ITS2 (5’—GCT GCG TTC TTC ATC GAT GC—3’; compare Wagner et al. 2019). Raw sequence reads were deposited at https://www.ncbi.nlm.nih.gov with BioProject accession number PRJNA1051341.

Sequence analysis was performed using the open source bioinformatic platform Quantitative Insight Into Microbial Ecology 2 (QIIME 2; Bolyen et al. 2019). Quality filtering was applied to raw sequence data and included the trimming of low-quality regions, removing short-length sequences, de-multiplexing and discarding sequences containing ambiguous bases and putative chimeras. Bacterial sequences were rarefied to 34321 reads per sample, and fungal sequences were rarefied to 50286 reads per sample. Remained high-quality sequences with more than 97% of similarity were joined into amplicon sequence variants (ASVs). Taxonomic assignment was performed with SILVA for bacterial (Quast et al. 2012) and UNITE for fungal sequences (Nilsson et al. 2019). Bacterial community structure was characterized at class level (with representability of classes more than 1%). Fungal community structure was characterized at family level (with representability of families more than 1%) after checking rarefaction (suppl. Figure S2).

### Data processing and statistical analysis

Soil parameters were standardized to have a mean of zero and a standard deviation of 1 according to formula

1 $$\left(\text{a} z-score\right)\text{zi}=(\text{xi}-\overline{\text{x} })/\text{s}$$

where z_i_ is standardized variable, x_i_ is a measured value, x̄ is mean value of a sample, and s is standard deviation of a sample.

Bacterial and fungal communities of each soil sample were characterized with community diversity indices (richness S, Shannon diversity H_SH_, Gini-Simpson H_GS_, Simpson dominance H_SD_, Berger-Parker H_BP_) based on the frequency of amplicon sequencing variants (ASVs).

Soil replicates within a variant of the experiment were compared to each other to determine the similarity/dissimilarity of microbial communities with Sørensen, Jaccard, and Bray–Curtis indices. Both for field and pot plants, mycorrhizosphere (MR) and bulk soil (BS) were considered.

Non-metric dimensional scaling (NMDS) based on the Bray–Curtis similarity index was used to visualize the similarity/dissimilarity of the experiment variants. The similarity percentages breakdown (SIMPER) procedure was applied to estimate the contribution of individual taxa in dissimilarity between compared groups of experiment variants based on the Bray–Curtis similarity index.

Taxonomical dataset and dataset of soil chemical parameters were split into subsets called herein as variants of the experiment: field plant MR, field plant BS, pot plant MR, control pot substrate, field plant sampling site (pooled data for field plant MR and corresponding BS). To determine, which parameters contribute the most to the structuring associated with plants bacterial and fungal communities, comparisons of variants of the experiment in different combinations were performed (Table 1).

**Table 1:** Diversity indices calculated for fungal and bacterial communities in different variants of the experiment

Variant	S	H_SD_	H_GS_	H_SH_	H_BP_
Fungal communities					
B_MR	138.67 ± 49.41	0.25 ± 0.06	0.75 ± 0.06	1.95 ± 0.49	0.38 ± 0.09
B_BS	176.00 ± 39.15	0.23 ± 0.07	0.77 ± 0.07	2.28 ± 0.31	0.41 ± 0.08
B_POT	133.00 ± 11.14	0.24 ± 0.25	0.76 ± 0.25	2.47 ± 0.88	0.36 ± 0.33
O_MR	162.00 ± 14.11	0.15 ± 0.05	0.85 ± 0.05	2.62 ± 0.37	0.29 ± 0.06
O_BS	172.33 ± 64.40	0.21 ± 0.12	0.79 ± 0.12	2.51 ± 0.41	0.38 ± 0.16
O_POT	145.50 ± 105.36	0.31 ± 0.29	0.69 ± 0.29	2.30 ± 1.31	0.48 ± 0.33
P_MR	182.67 ± 24.54	0.17 ± 0.08	0.83 ± 0.08	2.61 ± 0.34	0.33 ± 0.16
P_BS	261.00 ± 72.33	0.09 ± 0.05	0.91 ± 0.05	3.39 ± 0.60	0.21 ± 0.11
P_POT	142.00 ± 11.14	0.14 ± 0.03	0.86 ± 0.03	2.83 ± 0.12	0.27 ± 0.08
SUB	210.00 ± 17.78	0.04 ± 0.00	0.96 ± 0.00	3.87 ± 0.06	0.11 ± 0.01
Bacterial communities					
B_MR	434.67 ± 64.61	0.01 ± 0.00	0.99 ± 0.00	5.01 ± 0.17	0.06 ± 0.01
B_BS	393.67 ± 50.85	0.02 ± 0.00	0.98 ± 0.00	4.91 ± 0.12	0.06 ± 0.00
B_POT	312.67 ± 29.50	0.02 ± 0.00	0.98 ± 0.00	4.68 ± 0.09	0.08 ± 0.01
O_MR	576.67 ± 81.05	0.01 ± 0.00	0.99 ± 0.00	5.58 ± 0.13	0.03 ± 0.01
O_BS	755.67 ± 106.88	0.01 ± 0.01	0.99 ± 0.00	5.64 ± 0.19	0.04 ± 0.07
O_POT	385.00 ± 89.10	0.02 ± 0.00	0.98 ± 0.00	4.96 ± 0.31	0.06 ± 0.01
P_MR	597.33 ± 264.47	0.02 ± 0.01	0.98 ± 0.01	5.32 ± 0.64	0.08 ± 0.05
P_BS	582.33 ± 27.43	0.01 ± 0.00	0.99 ± 0.00	5.49 ± 0.10	0.04 ± 0.01
P_POT	280.67 ± 22.37	0.02 ± 0.00	0.98 ± 0.00	4.71 ± 0.11	0.05 ± 0.01
SUB	284.00 ± 21.00	0.03 ± 0.00	0.97 ± 0.00	4.52 ± 0.07	0.12 ± 0.01

S, richness; HSH, Shannon diversity index; HGS, Gini-Simpson index; HSD, Simpson dominance index; HBP, Berger-Parker index; B, birch; O, oak; P, pine; MR, mycorrhizosphere of field plants; BS, bulk soil; POT, mycorrhizosphere of pot plant; SUB, control pot substrate

To elucidate, which processes determine the assembly of bacterial and fungal community in mycorrhizosphere, the changes in microbial community structure along the simulated succession control pot substrate → pot plant MR → field plant MR were estimated*.* Impacts of age (0, unvegetated control pot substrate; initial, colonization of the substrate by a plant modelled in pots; primary, development of pioneer vegetation cover observed at the test field) as well as contents of total carbon, total nitrogen, total phosphorus, C/N ratio and pH, reflecting the state of soil development, were assessed.

Canonical correspondence analysis (CCA) and Spearman’s rank correlation analysis were performed to estimate the correlation of the most representative bacterial and fungal taxa, and diversity indices, with environmental variables. A *p*-value lower than 0.05 was considered as significant.

All multivariate analyses as well as Spearman’s rank correlation analysis were performed with an open-source software PAST 4.03. Check of significance of multivariate analyses results was performed with one-way analysis of similarities (ANOSIM) and one-way permutational analysis of variance (PERMANOVA). The significance was calculated by permutation of group membership (*N* = 9999). Bonferroni correction was used.

JASP 0.14.0.0 open-source software was used to perform Shapiro-Wilks’s test of normality. If the data were normally distributed, one-way ANOVA analysis with post hoc Tukey test was performed to identify significant differences between groups of values (relative abundance of taxa, diversity indices or environmental variables value). If Shapiro-Wilks’s test failed, non-parametric Kruskal–Wallis test with Bonferroni correction was used. Diversity indices were calculated with PAST 4.03. Community similarity indices (Sørensen, Jaccard, Bray–Curtis) were calculated for each variant with platform SPADE R online (Species Prediction And Diversity Estimation; Chao et al. 2005). One hundred bootstrap replications were applied. Calculation of mean values and standard deviation of the variables, calculation and depiction of relative abundance of the most representative taxa as well as differences between groups of variants was done with Microsoft Excel. Adjustment of output graphs produced in PAST 4.03 was performed for better representability with Adobe Illustrator.

## Results

### Microbial community composition

**Fig. 1:**
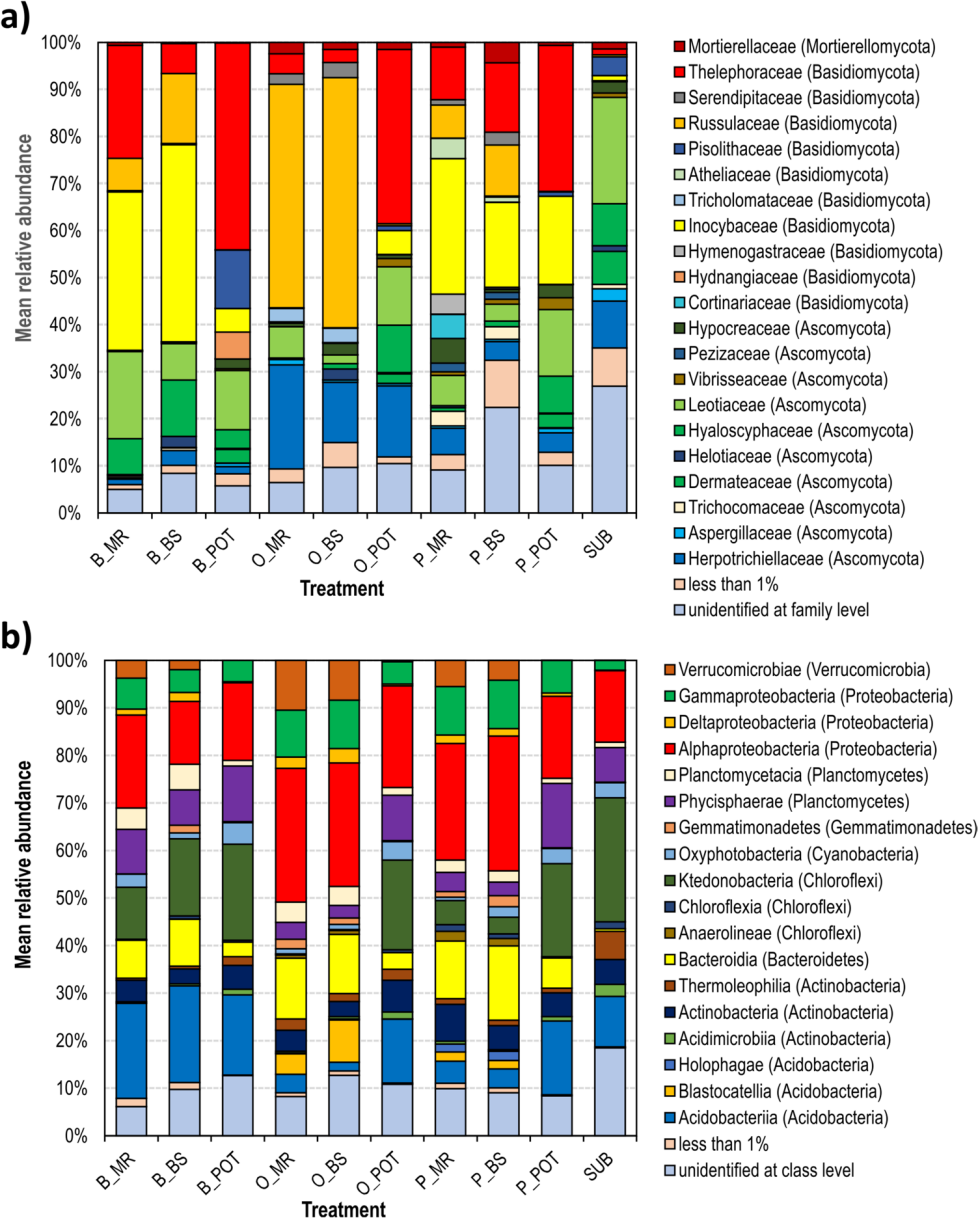
Structure of fungal (**a**) and bacterial (**b**) communities in different variants of the experiment. Fungal families and bacterial classes with a relative abundance of more than 1% are shown. B, birch; O, oak; P, pine; MR, mycorrhizosphere of field plant; BS, bulk soil; POT, mycorrhizosphere of pot plant; SUB, control pot substrate

The fungal as well as bacterial/archaeal communities were identified by ITS/16S rDNA community sequencing (Fig. 1). As might be expected, the ectomycorrhizosphere showed a lower richness and higher evenness compared to the surrounding bulk soil (Table 1, suppl. Tables S3-S4 Kruskal–Wallis test showing significance of differences). Nevertheless, the bulk soil showed, like the mycorrhizosphere, highest prevalence of fungal species for the ectomycorrhizal *Thelephoraceae*, *Inocybaceae* and *Russulaceae* (suppl. Figure S3). The species composition among the basidiomycete genera and their occurrence in all variants confirmed their dominance, which was also true for the unplanted pots. However, all pot experiments showed a lower diversity and different mycobiome composition (suppl. Table S5). In combination with *Leotiaceae* and *Herpotrichiellaceae,* ectomycorrhizal basidiomycetes explained up to 85% of the observed diversity (suppl. Table S6).

The most representative bacterial classes among all variants were *Alphaproteobacteria, Acidobacteria, Ktedonobacteria, Bacteroidia, Gammaproteobacteria* and *Phycisphaerae* (suppl. Figure S4). All together, they contributed from 59 to 80% of the bacterial community (suppl. Table S7). As expected, field variants showed highest richness and diversity while bacterial communities in pot soil were less even (compare Table 1). Bacterial communities within one tree species in field variants were less similar in diversity compared to pot plants of the same species (suppl. Table S8).

### Plant specificity of microbial communities

**Fig. 2:**
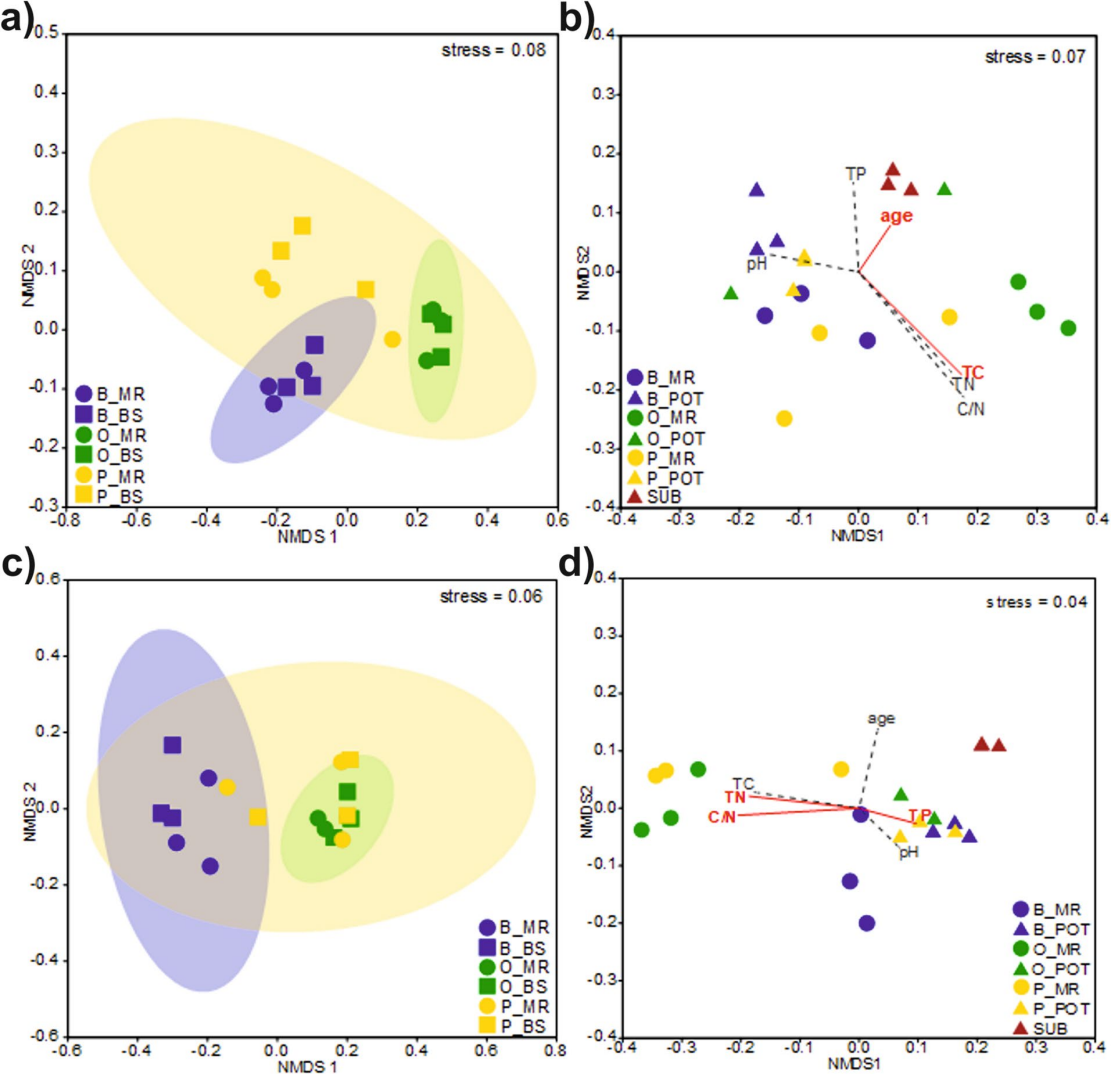
Non-metric dimensional scaling of field plants mycorrhizospheres and bulk soil based on the relative abundance of the most representative fungal families (**a**) and of fungal communities along successional stages (**b**), as well as of field mycorrhizospheres and bulk soil based on the relative abundance of the most representative bacterial classes (**c**) and of bacterial communities along successional stages (**d**). B, birch; O, oak; P, pine; MR, mycorrhizosphere of field plants; BS, bulk soil; POT, mycorrhizosphere of pot plants; SUB, control pot substrate; TC, total carbon content of soil; TN, total nitrogen content of soil; C/N, C/N ratio; TP total phosphorus content of soil; age: 0, unvegetated control pot substrate; initial, colonization of the substrate by a plant modelled in pots; primary, development of pioneer vegetation cover observed at the test field. Parameters with a significant effect are shown in red color

Three different tree species at the test field shaped their respective mycorrhizospheres shown by NMDS analysis (Fig. 2A), with a distinct separation of fungal communities and ectomycorrhizal basidiomycetes often being host specific. ANOSIM test (*R* = 0.79, *p* < 0.05) confirmed significant ordination of fungal communities with dissimilarity in fungal community of pairwise comparison between birch and oak field sites by *R*-value of 1.00 (suppl. Table S9). PERMANOVA test (*F* = 11.42, *p* < 0.05) confirmed this significance as well (suppl. Table S10). SIMPER analysis revealed that the most contributing to the dissimilarity between field sampling sites taxa were *Russulaceae, Inocybaceae, Thelephoraceae, Herpotrichiellaceae* (suppl. Table S11). *Russulaceae* and *Herpotrichiellaceae* were prominent especially in the field samples of oaks, and *Inocybaceae* with birches (Fig. 1A). These also slightly differentiated mycorrhizosphere from bulk soil. As with the field plant mycorrhizospheres, fungal communities inhabiting bulk soil were dominated by the same fungal families. Permutation analysis (*N* = 999, *p* = 0.03) showed that age (*p* = 0.011) and TC (*p* = 0.025) significantly determined ordination of fungal communities along the successional mycorrhizosphere development (see Fig. 2B), but significance was not confirmed by ANOSIM and PERMANOVA (see suppl. Tables S12-S15). Pairwise comparison of the variants revealed significant differences in all diversity indices only between control pot substrate and pot pines mycorrhizospheres (Table 1, suppl. Figure S5). Separation was seen between field sites of different trees for fungi, which has not been observed and not significantly determined in diversity indices for the bacterial community shown by classes in Fig. 1B and Table 1. But some genera, e.g., *Bradyrhizobium, Mesorhizobium* and higher abundances of *Sphingomonas* (within *Alphaproteobacteria*) combined with *Streptomyces* and *Kineosporia* (within *Actinobacteria*) were found in oak and pine field sites, and *Acidothermus* of *Actinobacteria* was prominent in birch field sites (suppl. Figure S4). Distinct separation of bacterial communities in one cluster of oak and pine (1 and 3) field sites and a second cluster formed in birch and pine (2) field sites also showed NMDS (Fig. 2C) with confirmed significance by ANOSIM, PERMANOVA and SIMPER (suppl. Tables S16-S18).

The pot plant communities were separated from unplanted control soil, hence showing an influence on fungal community selection. The bacteria separated on genus level show some influence, e.g., within the *Planctomycetaceae* (suppl. Figure S4). Therefore, it can be deduced that in general, the mycobiome was the main factor determining the bacterial microbiomes. The relative abundance of *Acidobacteria* in field birch mycorrhizospheres significantly exceeded those in oak or pine mycorrhizospheres, while pot plants and their fungal partners led to enrichment of *Acidobacteria, Alphaproteobacteria, Bacteroidia* and *Ktedonobacteria*. Hence, plant influence specifically on the fungal mycobiome in the ectomycorrhizosphere could be shown.

### Influence of soil parameters

**Fig. 3:**
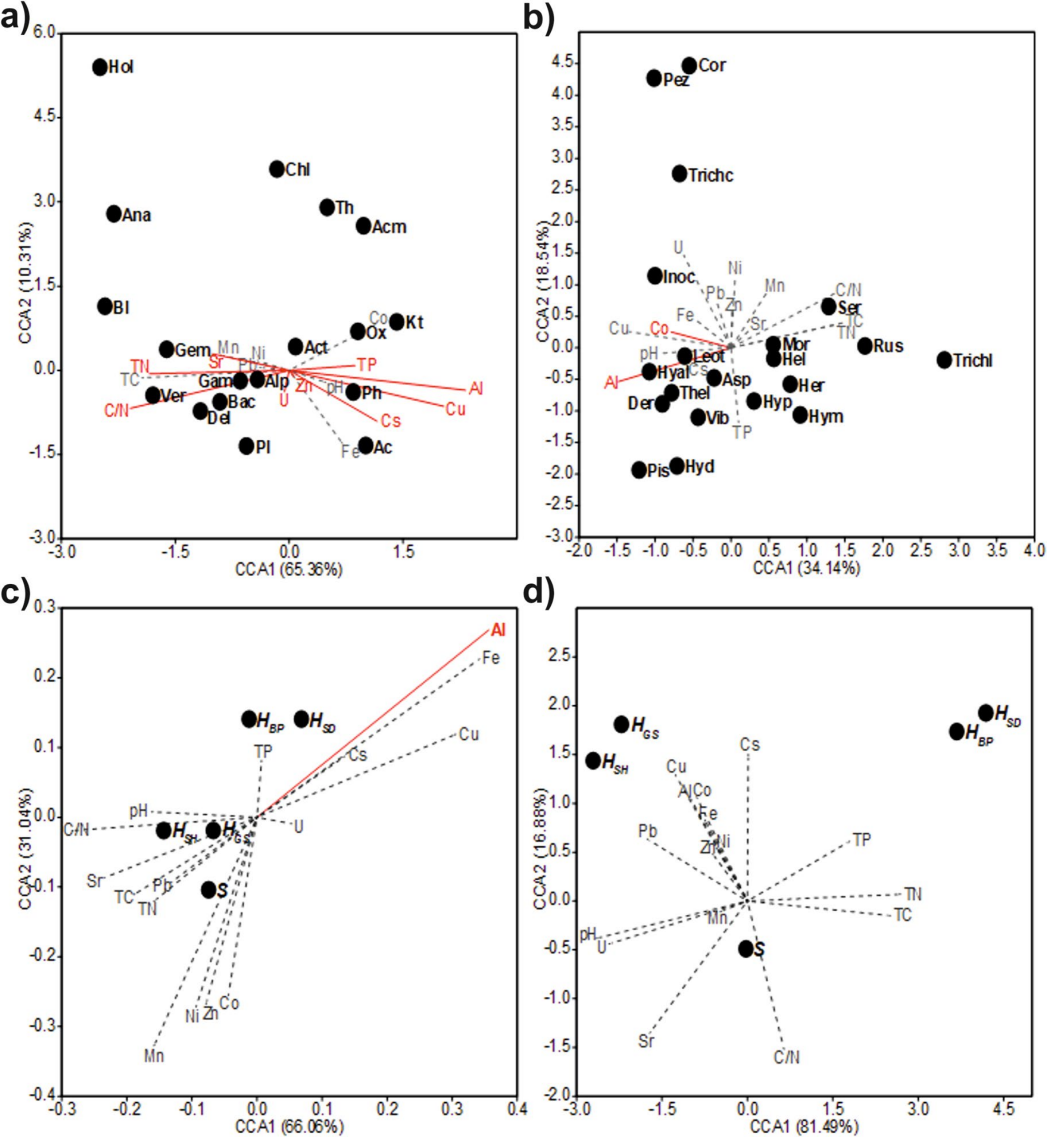
Canonical correspondence analysis biplot representing the correlation between soil parameters and the most representative bacterial classes (**a**), and most representative fungal families (**b**), the correlation between soil parameters and the bacterial community diversity indices (**c**), and fungal community diversity indices (**d**). Ac, *Acidobacteria;* Acm, *Acidimicrobia;* Act, *Actinobacteria;* Alp, *Alphaproteobacteria;* Ana, *Anaerolineae;* Bac, *Bacteroidia;* Bl, *Blastocatellia;* Chl, *Chloroflexia;* Del, *Deltaproteobacteria;* Gam, *Gammaproteobacteria;* Gem, *Gemmatimonadetes;* Hol, *Holophagae;* Kt, *Ktedonobacteria;* Ox, *Oxyphotobacteria;* Ph, *Phycisphaerae;* Pl, *Planctomycetacea;* Th,– *Thermoleophilia;* Ver, *Verrucomicrobiae; S,* richness; H_SH_, Shannon diversity index; H_GS,_ Gini-Simpson diversity; H_SD_, Simpson dominance index; H_BP_, Berger-Parker index; Asp, *Aspergillaceae;* Ath, *Atheliaceae;* Cor, *Cortinariaceae;* Der, *Dermateaceae;* Hel, *Helotiaceae;* Her, *Herpotrichiellaceae;* Hyal, *Hyaloscyphaceae;* Hyd, *Hydnangiaceae;* Hym, *Hymenogastraceae;* Hyp, *Hypocreaceae;* Inoc, *Inocybaceae;* Leot, *Leotiaceae;* Mor, *Mortierellaceae;* Pez, *Pezizaceae;* Pis, *Pisolithaceae;* Rus, *Russulaceae;* Ser, *Serendipitaceae;* Thel, *Thelephoraceae;* Trichc, *Trichocomaceae;* Trichl, *Tricholomataceae;* Vib, *Vibrisseaceae*. Soil parameters with a significant effect are shown in red color

The natural stands of young trees varied with respect to soil parameters, see details in Bogdanova et al. (2023). To see, if soil is more important to structure the ectomycorrhizosphere communities than the trees, the same potting soil with the three different tree species was used. Indeed, the bacterial community strongly corresponded with Al, Cu and Zn as well as Sr and Cs (Fig. 3). At the same time, total N, P, and the C/N ratio significantly correlated with bacterial community structure. Permutation analysis (*N* = 999, *p* = 0.001) showed significance of TN (*p* = 0.028), TP (*p* = 0.017), and C/N (*p* = 0.001), but not the stage of succession, in ordination of bacterial communities along the successional mycorrhizosphere development (see Fig. 2D). ANOSIM (Tables S19-S20) and PERMANOVA (Tables S21-S22) tests did not confirm significance of ordination based on the successional gradients.

Bacterial classes, which contributed to the dissimilarity between sampling sites most, were significantly associated with soil characteristics typical for this site. *Acidobacteria,* abundant at the birch sampling site, showed a negative relationship with Mn, Pb, and Sr and a positive one with the contents of Cu, Fe, and Cs (Table 2). *Ktedonobacteria* positively correlated with Al, Cu, U, and Fe. *Alphaproteobacteria*, dominant at the oak and pine sampling sites, positively correlated with C/N ratio, Sr and Mn, and negatively associated with Al, Cu, and Fe. Overall, the bacterial community diversity indices showed Al concentration, total C and C/N ratio to be major determinants (Table 3).

**Table 2:** Coefficients of correlation between soil characteristics and fungal families as well as bacterial classes with relative abundance higher than 1%

	Al	Co	Cu	Fe	Mn	Ni	Pb	Sr	Zn	Cs	U	TC	TN	C/N	TP	pH
Fungi																
Her	**−0.46**	−0.01	**−0.45**	−0.32	**0.43**	0.17	0.04	0.30	0.18	−0.36	**−0.58**	0.20	0.24	0.26	0.11	**−0.42**
Asp	−0.02	**0.70**	0.10	−0.04	**0.43**	0.20	0.02	0.18	**0.60**	−0.20	**−0.39**	**−0.42**	**−0.42**	−0.36	0.23	−0.04
Trichc	0.14	0.13	0.33	0.22	−0.12	−0.20	0.02	−0.11	−0.11	−0.03	**0.59**	−0.07	−0.04	−0.04	**−0.55**	0.18
Der	**0.50**	**0.77**	0.31	0.16	−0.04	−0.12	−0.10	−0.11	**0.39**	0.18	0.02	**−0.73**	**−0.75**	**−0.72**	0.19	0.23
Hel	0.14	0.18	−0.04	0.28	−0.15	−0.30	**−0.46**	−0.31	−0.13	−0.04	−0.31	**−0.37**	−0.32	**−0.38**	**0.41**	−0.34
Hyal	**0.74**	0.25	**0.57**	**0.72**	**−0.66**	**−0.64**	**−0.84**	**−0.85**	0.00	**0.44**	−0.13	**−0.58**	**−0.50**	**−0.63**	**0.38**	−0.19
Leot	**0.63**	**0.58**	**0.52**	**0.57**	−0.29	−0.31	**−0.45**	**−0.54**	0.32	**0.55**	−0.02	**−0.57**	**−0.52**	**−0.64**	0.12	0.03
Vib	0.17	0.24	−0.20	−0.25	0.13	−0.10	0.17	0.26	0.02	−0.15	0.20	**−0.47**	**−0.49**	−0.33	−0.15	0.33
Pez	−0.17	0.17	0.16	−0.17	**0.42**	**0.38**	**0.51**	**0.51**	0.20	−0.24	**0.53**	0.07	0.00	0.18	**−0.53**	**0.38**
Hyp	−0.01	0.14	−0.24	−0.25	0.22	−0.08	0.01	0.20	0.02	−0.35	−0.15	−0.32	−0.30	−0.25	0.11	0.02
Cor	**−0.49**	−0.05	−0.35	**−0.40**	**0.53**	0.15	0.24	**0.45**	0.11	**−0.42**	−0.05	0.13	0.17	0.23	−0.18	−0.08
Hyd	**0.37**	0.29	0.34	**0.54**	−0.23	**−0.41**	**−0.63**	**−0.53**	−0.09	0.15	−0.04	−0.34	−0.27	**−0.38**	0.25	−0.32
Hym	−0.15	−0.29	**−0.50**	**−0.44**	0.23	−0.03	0.34	**0.41**	**−0.43**	**−0.40**	0.29	−0.01	−0.02	0.12	**−0.49**	0.20
Inoc	**0.42**	−0.13	**0.53**	**0.41**	**−0.53**	**−0.38**	−0.08	**−0.39**	−0.23	0.19	**0.70**	−0.07	−0.07	−0.13	**−0.37**	0.21
Trichl	**−0.68**	−0.33	**−0.45**	−0.30	0.33	0.12	−0.04	0.27	−0.15	**−0.57**	**−0.41**	0.37	**0.41**	**0.47**	0.13	**−0.42**
Pis	**0.53**	**0.40**	0.13	0.32	−0.30	**−0.51**	−0.36	**−0.41**	0.14	0.28	−0.19	**−0.74**	**−0.74**	**−0.72**	0.36	0.04
Rus	**−0.54**	**−0.55**	**−0.40**	−0.10	0.07	−0.03	−0.15	0.03	−0.31	−0.19	−0.36	**0.60**	**0.67**	**0.58**	0.00	**−0.50**
Ser	**−0.64**	−0.24	**−0.39**	**−0.60**	**0.59**	**0.52**	**0.54**	**0.70**	0.11	**−0.47**	−0.03	**0.57**	**0.53**	**0.65**	−0.23	−0.02
Thel	**0.36**	0.02	0.21	0.02	−0.19	0.08	0.22	0.05	0.09	0.31	0.32	−0.18	−0.32	−0.13	0.01	**0.61**
Mor	**−0.51**	−0.21	**−0.49**	**−0.48**	**0.41**	0.26	0.28	**0.43**	−0.03	**−0.45**	−0.20	0.32	0.31	**0.42**	−0.27	−0.17
Bacteria																
Ac	0.72	0.17	**0.76**	**0.76**	**−0.62**	−0.39	**−0.62**	**−0.78**	0.15	**0.71**	0.08	−0.38	−0.26	**−0.58**	0.26	0.04
Bl	**−0.89**	0.19	**−0.60**	**−0.71**	**0.63**	**0.60**	0.43	**0.52**	0.22	**−0.55**	−0.46	**0.65**	**0.56**	**0.63**	0.09	−0.33
Hol	**−0.63**	0.10	**−0.58**	**−0.64**	**0.70**	**0.63**	**0.55**	**0.55**	0.03	**−0.61**	−0.30	0.32	0.21	0.38	−0.11	−0.27
Act	0.22	0.21	−0.18	−0.14	0.46	0.34	**0.49**	0.46	0.01	0.06	0.38	−0.22	−0.29	−0.06	**−0.60**	**0.58**
Th	**−0.65**	**0.49**	−0.25	−0.44	**0.68**	**0.65**	**0.53**	0.45	**0.52**	−0.10	−0.35	0.46	0.43	0.29	0.03	−0.13
Bac	−0.43	0.02	−0.42	**−0.49**	0.45	0.26	**0.55**	**0.51**	0.23	−0.14	−0.14	0.23	0.13	0.34	−0.29	0.13
Ana	**−0.63**	−0.01	**−0.77**	**−0.84**	**0.60**	**0.56**	**0.62**	**0.70**	−0.24	**−0.70**	−0.07	0.32	0.22	0.46	−0.24	−0.04
Chl	−0.12	−0.38	−0.27	**−0.58**	0.04	0.03	**0.56**	**0.49**	−0.35	−0.47	0.45	−0.02	−0.11	0.11	−0.40	0.03
Kt	**0.85**	−0.13	**0.66**	**0.52**	**−0.48**	**−0.49**	−0.13	−0.28	0.01	0.44	**0.60**	**−0.55**	**−0.47**	**−0.58**	−0.29	0.34
Gem	**−0.55**	−0.11	**−0.57**	**−0.71**	0.20	0.28	**0.55**	**0.49**	−0.13	−0.30	0.01	0.43	0.34	0.41	−0.12	−0.15
Ph	**0.81**	0.00	**0.78**	**0.79**	**−0.49**	**−0.48**	**−0.49**	**−0.59**	0.14	**0.57**	0.31	**−0.50**	−0.40	**−0.62**	−0.01	0.13
Pl	0.28	**−0.50**	0.15	**0.53**	**−0.48**	**−0.76**	**−0.66**	**−0.61**	−0.34	0.23	−0.16	−0.17	−0.02	−0.23	0.34	−0.26
Alp	**−0.59**	0.04	**−0.53**	**−0.47**	**0.48**	0.45	0.44	**0.59**	−0.05	−0.43	−0.10	0.46	0.29	**0.74**	−0.04	0.04
Del	**−0.72**	−0.26	**−0.48**	−0.40	0.03	0.06	−0.01	0.06	−0.28	**−0.49**	−0.38	**0.53**	**0.52**	0.38	0.29	**−0.63**
Gam	**−0.63**	0.17	**−0.62**	**−0.74**	**0.68**	**0.56**	**0.62**	**0.76**	0.03	**−0.51**	−0.09	0.08	0.02	0.30	−0.19	0.23
Ver	**−0.75**	−0.17	**−0.68**	−0.40	0.42	0.18	0.04	0.21	−0.02	−0.37	**−0.60**	**0.52**	**0.47**	**0.60**	0.29	−0.42

Asp, *Aspergillaceae;* Ath, *Atheliaceae;* Cor, *Cortinariaceae*; Der, *Dermateaceae;* Hel, *Helotiaceae;* Her, *Herpotrichiellaceae;* Hyal, *Hyaloscyphaceae;* Hyd, *Hydnangiaceae;* Hym, *Hymenogastraceae;* Hyp, *Hypocreaceae;* Inoc, *Inocybaceae;* Leot, *Leotiaceae;* Mor, *Mortierellaceae;* Pez, *Pezizaceae;* Pis, *Pisolithaceae;* Rus, *Russulaceae;* Ser, *Serendipitaceae;* Thel, *Thelephoraceae;* Trichc, *Trichocomaceae;* Trichl, *Tricholomataceae;* Vib, *Vibrisseaceae;* Ac, *Acidobacteria*; Act, *Actinobacteria;* Alp, *Alphaproteobacteria;* Ana, *Anaerolineae;* Bac, *Bacteroidia;* Bl, *Blastocatellia;* Chl, *Chloroflexia;* Del, *Deltaproteobacteria;* Gam, *Gammaproteobacteria;* Gem, *Gemmatimonadetes;* Hol, *Holophagae;* Kt, *Ktedonobacteria;* Ph, *Phycisphaerae;* Pl, *Planctomycetacea;* Th, *Thermoleophilia;* Ver, *Verrucomicrobiae;* bold print represents significant correlation (*p* < 0.05)

**Table 3:** Coefficients of correlation between soil characteristics and fungal community as well as bacterial community diversity indices

Soil characteristics	S	H_SD_	H_GS_	H_SH_	H_BP_
Fungi					
Al	−0.24	−0.22	0.22	−0.16	−0.35
Co	−0.24	−0.18	0.18	−0.08	−0.22
Cu	−0.04	−0.18	0.17	−0.01	−0.26
Fe	−0.21	0.01	0.00	−0.18	−0.15
Mn	0.26	−0.08	0.08	0.24	−0.01
Ni	0.19	−0.03	0.02	0.10	0.00
Pb	**0.49**	−0.19	0.18	0.31	−0.12
Sr	**0.44**	−0.23	0.22	0.38	−0.14
Zn	−0.06	−0.03	0.04	−0.09	−0.02
Cs	**−0.47**	0.12	−0.11	**−0.47**	0.00
U	**0.42**	−0.37	0.35	0.34	−0.36
TC	0.26	0.25	−0.26	0.05	0.31
TN	0.19	0.37	−0.38	−0.04	**0.43**
C/N	0.35	0.06	−0.07	0.25	0.12
TP	**−0.54**	**0.43**	**−0.42**	**−0.48**	0.34
pH	0.04	**−0.55**	**0.54**	0.29	**−0.56**
Bacteria					
Al	**−0.76**	**0.51**	**−0.51**	**−0.74**	0.22
Co	−0.04	0.11	−0.11	0.00	0.10
Cu	**−0.50**	0.38	−0.38	−0.46	0.12
Fe	**−0.61**	0.36	−0.36	**−0.52**	0.17
Mn	0.36	−0.35	0.35	0.44	−0.09
Ni	0.40	−0.29	0.29	0.43	−0.15
Pb	0.25	−0.30	0.30	0.28	−0.26
Sr	0.33	−0.24	0.24	0.30	−0.11
Zn	−0.03	−0.07	0.07	0.13	−0.03
Cs	**−0.63**	0.35	−0.35	**−0.51**	0.27
U	−0.40	0.39	−0.39	**−0.52**	0.24
TC	**0.54**	**−0.50**	**0.50**	**0.61**	−0.44
TN	0.44	−0.35	0.35	**0.48**	−0.31
C/N	**0.58**	**−0.65**	**0.65**	**0.65**	**−0.56**
TP	0.13	−0.10	0.10	0.16	−0.16
pH	−0.38	0.36	−0.36	−0.44	0.39

S, richness; H_SH_, Shannon diversity index; H_GS_, Gini-Simpson index; H_SD_, Simpson dominance index; H_BP_, Berger-Parker index; bold print represents significant correlation (*p* < 0.05)

The fungal families responded specifically to Al and Co (compare Fig. 3 and Table 3). While *Thelephoraceae* and *Herpotrichiellaceae* were found in all variants of the experiment, soil pH seemed to be a major determinant for their prevalence. The ascomycete family *Leotiaceae*, highly presented at the birch sampling site and in all pot variants, positively correlated with Al, Co, Cu, Fe, Cs, and negatively with Pb, Sr, total C, total N, and C/N ratio. Thus, fungal community structure was more likely to be influenced by the plant, while the bacterial community, in addition to being dependent on fungi present, showed a stronger impact of soil parameters.

## Discussion

### The (mycor)rhizosphere effect

Post-mining areas are generally characterized by high soil heterogeneity, and, initially, a primary succession is observed on disturbed substrates (Iordache et al. 2009). In such an environment, the activity of the root-associated microbes might especially profit from root exudates, or a rhizosphere effect (Badri and Vivanco 2009; Yang et al. 2023). In addition, the effects of ectomycorrhizal fungi extending the area of tree-derived deposition of nutrients and mediating heavy metal and water transport along their hyphae (Traxler et al. 2022) was expected to significantly contribute to a “mycorrhizosphere effect.” With this study, such a mycorrhizosphere effect could be seen with the fungal community being determined by tree species and the bacterial community largely dependent on the composition of the mycobiome present (Shi et al. 2023). However, if re-distributed through a fungal network, this line of argument would no longer be valid in a natural setting, specifically with mycorrhizal fungi showing long-range exploitation types.

Moreover, the association with fungi is known to change the extent and composition of root exudates (Meier et al. 2013; Uroz et al. 2016). This mycorrhizosphere effect was most visible in the current investigation when comparing the unplanted control soil with the planted counterparts. The bacterial microbiome in these pots showed a strong impact of high Al contents combined with low nutrient availability. In accordance with such a mycorrhizosphere effect, the presence of mycorrhizal fungi facilitated changes in bacterial microbiome in the planted pots. The distinction between planted and unplanted pots was clearly seen, which supports the contribution of the mycobiome to a mycorrhizosphere effect.

### Ectomycorrhizal taxa dominated the mycorrhizosphere mycobiomes

Unidentified at genus level, *Thelephoraceae* contributed the most to the fungal diversity. *Thelephora terrestris* would be the most widely found species, which is forming ectomycorrhiza (Cabrera-Ariza et al. 2023). Correlation analysis revealed that only pH value had a strong positive effect on the presence of *Thelephoraceae*. This family encompasses pioneer fungi and contributes to an ECM-resistant propagule bank in disturbed soils (Kałucka and Jagodziński 2017). *Russula*, *Inocybe* and *Lactarius* were present in the planted pots and field sites in high amounts among the mycobiome members as well. As these also are ectomycorrhizal members, host interaction clearly dominated the mycobiomes.

*Inocybe* is attributed to early successional stages and has been identified as dominant genus with field birch and pine (Ishida et al. 2009). In contrast, *Russula* was more prevalent in a more advanced successional habitat, the field oak sampling site (Kolaříková et al. 2017). Correlation analysis revealed strong associations between *Russulaceae* and the content of total carbon and total nitrogen, which confirms that the dynamics of *Russula* was determined by vegetation succession. The *Lactarius* group exhibits narrow host range, which is also a sign for a later stage of succession with more specific interactions (Parladé et al. 2007). Examples would include *Lactarius pubescens* on birch, *Lactarius quietus* with oak or *Lactarius deliciosus* specific for pine. Thus, the naturally grown young trees already profited from the grown trees in direct vicinity that spawned this natural progression of forest development on a formerly barren soil.

### (Heavy) metal impact on the mycorrhizosphere bacteria

Early stages of succession microbiomes are thought to be predominantly determined by abiotic conditions including low nutrient availability, especially with the low pH and presence of toxic metals in post-mining areas (Šnajdr et al. 2013; Pietrzykowski 2019). As microorganisms may change metal availability, their impact in post-mining areas is expected to increase in the mycorrhizosphere (Wang et al. 2023; Agarwal et al. 2023; Lammel et al. 2018). Here, the elevated Al concentration was among the most affecting soil parameters. *Ktedonobacteria (Chloroflexaceae)* were enriched, especially in the pot experiment (Wen et al. 2023). Canonical correspondence analysis confirmed by correlation analysis revealed association of this taxon with high Al, Cu, and U contents and low nutrients, which has been seen before (Chang et al. 2011; Epelde et al. 2015).

Mechanisms for increased Al as well as heavy metal tolerance like release of siderophores might explain the enrichment of this group of organisms (Dimkpa et al. 2009). In contrast, the mycorrhizal fungal mycobiome responds rather to host interactions than being dependent on variable abiotic conditions (Kolaříková et al. 2017; Martínez-García et al. 2015).

Linked to the low nutrient availability, nitrogen fixation may be important. Free-living diazotrophs include *Cyanobacteria*, which also have been enriched in our experiments. In addition, the cluster *Burkholderia-Caballeronia-Paraburkholderia* (*Gammaproteobacteria*) was more abundant in pot plants’ mycorrhizospheres than the corresponding field plants and *Bradyrhizobium*, *Mesorhizobium* and *Allorhizobium-Neorhizobium-Pararhizobium-Rhizobium* (*Alphaproteobacteria)* showed higher presence in oaks and pine field sites than in the corresponding pots. *Burkholderia* as well as the mentioned rhizobia are commonly present in rhizospheres and also able to fix N_2_ (Puri et al. 2020; Hsu et al. 2018). *Alphaproteobacteria* was one of the most abundant bacterial taxa, with prominent *Sphingomonas* known to promote plant growth (Asaf et al. 2020; Luo et al. 2019). Therefore, the presence of *Sphingomonas* might be beneficial for the plants as well in terms of their growth under unfavourable abiotic conditions.

### The combination of pot and field experiments helps to mimic succession

The present study attempted to separate the impact of soil chemical parameters from the plant influence, therefore the experimental consisted of two parts: 1) the study of field sites of, presumably, the same age but at different levels of development, and 2) a pot experiment, which modelled an initial stage of succession with similar abiotic conditions for all trees.

The glasshouse experiment demonstrated that 2 years were not enough to establish plant species-specific mycorrhizospheres. Ordination plots did not show a tendency to distinguish either bacterial or fungal communities based on their associations with particular tree species. This result supports an assumption that observed at the test field differences in microbial communities, initially attributed to the tree species, were determined predominantly by site identity. Several studies demonstrated similar observations and showed that different plant species might shape identical in the structure microbial communities at the early stages of ecosystem development. Arctic environment plants belonging to various species were associated with similar fungal communities suggesting that symbiosis with fungi-generalists may facilitate more efficient colonization of new habitats (Botnen et al. 2014). Nunan et al. (2005) demonstrated that plant species did not impact bacterial community composition in grassland soil. In contrast, environmental factors were crucial for explaining bacterial community structure in the rhizoplane of grasses. Moreover, Tscherko et al. (2005) observed plant species’ effect on the composition and activity of rhizosphere microorganisms only after 43 years of succession in a recently deglaciated alpine terrain.

Although the statistical tests did not confirm the significance of all discussed above patterns, the received results can be considered as expected trends. High spatial heterogeneity typical for post-mining areas could lead to high inter-variant variability and explain why the comparison of the variants did not attain statistical significance. On the other hand, rarefaction curves reached plateau both for bacterial and fungal communities, suggesting that microbial populations of each subsample were fully represented, and the most abundant as well as rare species were covered by sequence. The increase of sampling can overcome this inconclusiveness related to the statistical insignificance of results. Thus, characteristics of community diversity based on larger sampling should theoretically approach those characteristics as estimated for the entire population (Bolyen et al. 2019). On the other hand, one should consider the fact that former mines are highly variable environments; therefore, the increase in sampling might not necessarily improve their representativeness.

### Conclusions for afforestation in post-mining conditions

With this work, we could prove our hypotheses, and drivers for microbial community composition could be separated. The afforestation of post-mining landscapes which are not suitable for agricultural use is a preferrable option that would provide recreational value and, in addition, prevent erosion of substrates covering former heap sides. The forestry use then also may cover some of the costs for remediation (Sui et al. 2023).

In order to establish a new forest on soil that is low in nutritional value, specifically N and P, does not contain substantial C_org_ and is characterized often by low water holding capacity as well as (heavy) metal loads, requires special measures. The use of ectomycorrhiza and a bacterial consortium to support tree growth is one measure that could improve tree establishment (Shi et al. 2023). For this reason, we evaluated whether the soil or the tree species is the main driver for the mycorrhizosphere microbiome composition. Using three tree species typical for boreal forests, we could show that the mycobiome is selected from fungal propagules present—or which can be supplied as inoculum—at the site specific for each tree species. The prevalence of ascomycetes in the unplanted substrate is replaced by ectomycorrhizal basidiomycetes, allowing for better tree nutrition and water provision of the trees. The basidiomycete fungi were less strongly affected by the soil as compared to the bacterial members of the microbiome.

The fungal community selected for certain groups of bacteria. However, the influence of the soil and its bioavailable metal content were prominent in our experiments. Therefore, an inoculation with bacteria from non-contaminated areas seems less promising. Rather, we can conclude that if inoculation is necessary, it should use strains isolated from pot experiments with the autochthonous substrate, and the tree species to be planted. This would also be in agreement with earlier observations using herbaceous plants (Kothe and Büchel 2014).

## Supplementary Information

Below is the link to the electronic supplementary material.

Supplementary file1 (PDF 1.81 MB)

## Data Availability

All data are provided with this submission.
