# Supplementary material for: Drivers of fungal and bacterial communities in ectomycorrhizospheres of birch, oak, and pine in a former uranium mining site, Ronneburg, Germany
Source: Environ Sci Pollut Res Int. 2025 Apr 2;32(17):10786–99. doi: 10.1007/s11356-025-36330-6 (PMC12014720; doi:10.1007/s11356-025-36330-6)
Supplement: Supplementary file 1 — Supplementary file1 (PDF 1.81 MB) [file 11356_2025_36330_MOESM1_ESM.pdf]

Supplementary materials

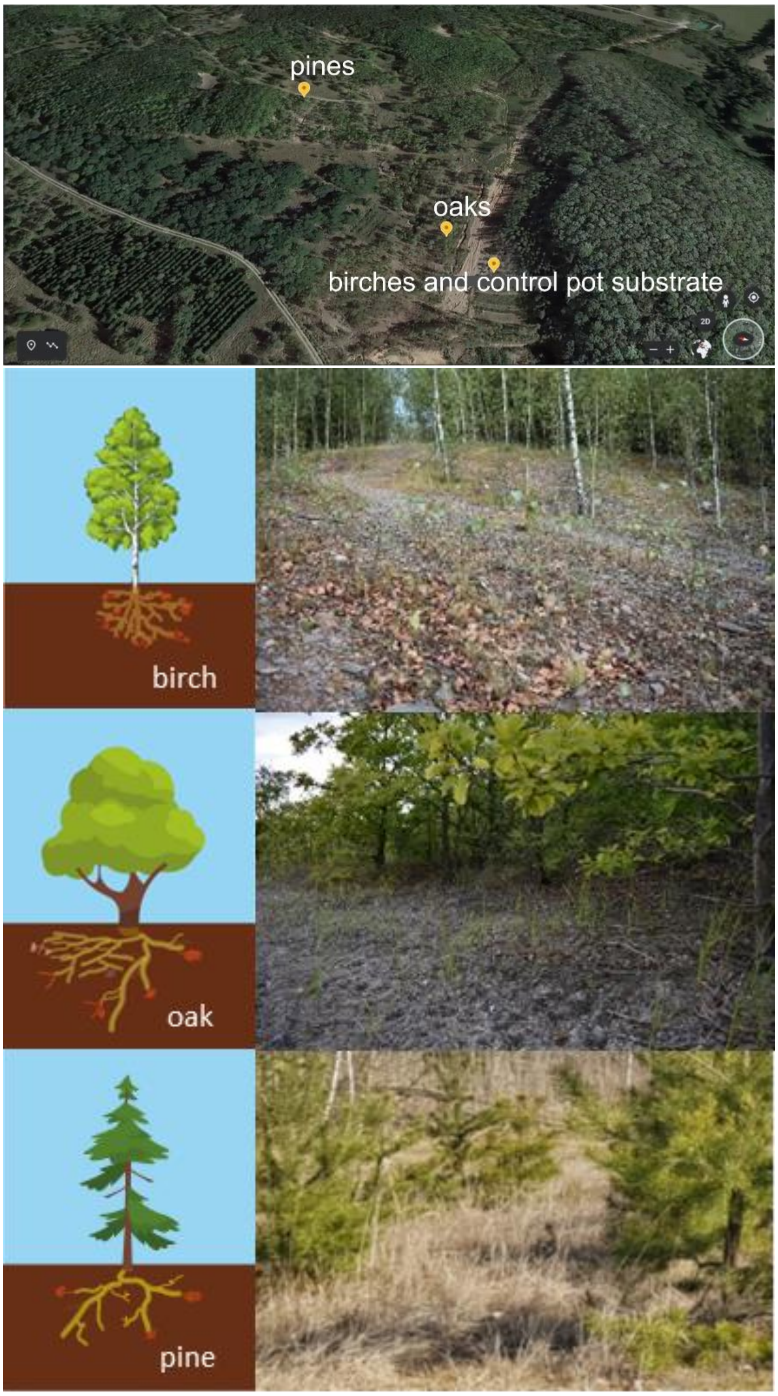

Figure S1. Sampling sites of birch, oak and pine stands at Kanigsberg near Ronneburg in the former uranium mining area, Germany, including a map from Google Earth, WEB version from 2017.

Table S1. Selected soil chemical parameters in the different variants of the experiment (Bogdanova et al. 2023)

Variant	pH	TC (%)	TN (%)	TP (mg/kg)
B_MR	4.44 ± 0.32	0.87 ± 0.24	0.11 ± 0.01	669 ± 173
B_BS	3.75 ± 0.30	1.07 ± 0.05	0.13 ± 0.01	603 ± 198
B_POT	6.03 ± 0.36	0.39 ± 0.05	0.07 ± 0.00	769 ± 285
O_MR	3.54 ± 0.10	3.45 ± 0.35	0.31 ± 0.05	786 ± 127
O_BS	3.56 ± 0.09	2.60 ± 0.85	0.27 ± 0.07	802 ± 193
O_POT	5.32 ± 0.42	0.36 ± 0.01	0.07 ± 0.00	843 ± 100
P_MR	5.20 ± 1.47	0.87 ± 0.13	0.09 ± 0.01	347 ± 61
P_BS	6.21 ± 0.53	1.11 ± 0.44	0.10 ± 0.03	369 ± 37
P_POT	5.52 ± 0.22	0.37 ± 0.01	0.07 ± 0.00	718 ± 178
SUB	3.47 ± 0.01	0.33 ± 0.01	0.07 ± 0.00	801 ± 121

B, birch; O, oak; P pine; MR, mycorrhizosphere of field plant; BS, bulk soil; SUB, control pot substrate; TC, total carbon content; TN, total nitrogen content; TP total phosphorus content.

Table S2: Content of bioavailable metals (μg/g soil) in the different variants of the experiment (Bogdanova et al. 2023)

Variant	Al	Cd	Co	Cr	Cu	Fe	Mn	Mo	Ni	Pb	Sr	V	Zn	Cs	U
B_MR1	320.000	0.031	0.442	0.143	9.060	43.600	8.412	0.065	0.440	0.013	0.258	0.065	0.842	0.145	2.976
B_BS1	311.200	0.044	0.664	0.297	9.840	58.930	11.160	0.065	0.560	0.013	0.285	0.065	1.100	0.118	2.775
B_MR2	344.100	0.032	0.620	0.200	10.240	47.500	10.657	0.065	0.600	0.013	0.281	0.065	0.900	0.125	2.887
B_BS2	328.300	0.022	0.390	0.200	11.900	24.470	10.400	0.065	0.400	0.013	0.287	0.065	1.100	0.097	3.115
B_MR3	298.900	0.035	0.481	0.180	11.700	71.400	10.820	0.065	0.501	0.013	0.313	0.065	1.300	0.078	2.660
B_BS3	262.900	0.022	0.334	0.125	7.860	10.360	7.056	0.065	0.250	0.078	0.292	0.065	0.671	0.122	3.746
O_MR1	7.000	0.217	0.560	0.125	4.740	5.393	126.200	0.234	0.870	0.091	3.222	0.652	5.220	0.038	0.493
O_BS1	6.500	0.058	0.413	0.125	3.420	1.870	22.768	0.130	0.930	0.015	1.554	1.080	0.650	0.029	0.729
O_MR2	6.500	0.072	0.635	0.160	4.040	1.821	45.400	0.180	1.590	0.032	1.902	0.960	1.150	0.027	0.860
O_BS2	6.500	0.114	0.610	0.125	3.720	2.280	55.800	0.187	0.930	0.042	1.898	0.740	2.950	0.123	0.799
O_MR3	7.700	0.127	0.688	0.140	2.863	3.999	73.330	0.173	1.280	0.119	2.889	0.263	3.250	0.236	0.712
O_BS3	6.500	0.099	0.671	0.140	4.400	2.300	49.847	0.159	1.270	0.085	3.268	0.884	1.020	0.033	0.868
P_MR1	9.400	0.046	0.399	0.125	3.670	1.510	50.480	0.065	0.950	0.453	10.900	0.065	0.750	0.035	3.516
P_BS1	10.220	0.041	0.630	0.450	2.930	1.600	58.450	0.065	1.080	0.465	11.075	0.065	0.700	0.045	2.639
P_MR2	38.000	0.320	2.400	0.125	9.734	2.300	218.000	0.065	7.445	0.510	14.198	0.077	7.800	0.082	6.810
P_BS2	189.400	0.079	1.186	0.125	12.010	8.360	45.941	0.065	1.710	2.576	11.079	0.065	3.800	0.105	5.199
P_MR3	8.100	0.038	0.313	0.125	2.560	2.666	67.450	0.114	0.580	0.369	43.980	0.157	0.650	0.029	4.414
P_BS3	6.800	0.020	0.232	0.125	1.770	1.300	13.582	0.065	0.400	0.340	11.875	0.065	0.650	0.047	3.829
SUB	312.200	0.042	1.230	0.125	8.410	8.970	24.800	0.065	0.460	0.013	1.131	0.065	1.380	0.094	1.419

B, birch; O, oak; P pine; MR, mycorrhizosphere of field plant; BS, bulk soil; SUB, control pot substrate.

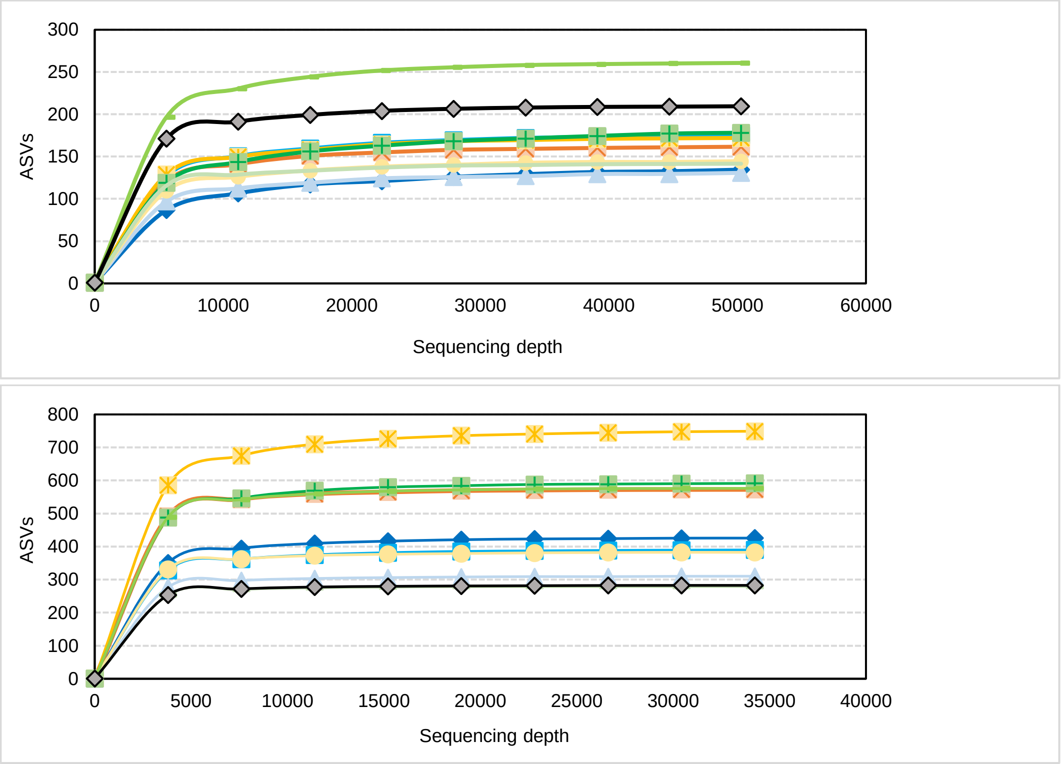

Figure S2. Rarefaction curves for fungal (upper) and bacterial sequences (down). ASV, amplicon sequence variant; B, birch (blue); O, oak (orange); P, pine (green); MR, mycorrhizosphere of field plant (dark color); BS, bulk soil (medium color); POT, mycorrhizosphere of pot plant (light color); SUB, control pot substrate (black).

Table S3. One-Way ANOVA, Kruskal-Wallis test, post hoc test output for significance of differences between variants of the experiment in diversity indices of bacterial communities.

Diversity Index	ANOVA						Kruskal-Wallis				Post Hoc comparisons						
	**Cases**	**Sum of Squares**	**df**	**Mean Square**	**F**	**p**	**Factor**	**Statistic**	**df**	**p**	**Variant of comparisons**		**Mean Difference**	**SE**	**t**	**p tukey**	**p bonf**
Richness	variant	2.521.500	1	2.521.500	0.746	0.436					B_MR	B_BS	-41.000	47.468	-0.864	0.436	0.436
	Residuals	13.519.333	4	3.379.833													
	variant	48.061.500	1	48.061.500	5.343	0.082					O_MR	O_ BS	179.000	77.442	2.311	0.082	0.082
	Residuals	35.983.333	4	8.995.833													
	variant	337.500	1	337.500	0.010	0.927					P_MR	P_BS	-15.000	153.513	-0.098	0.927	0.927
	Residuals	141.397.333	4	35.349.333													
	variant	22.326.000	1	22.326.000	8.851	0.041					B_MR	B_POT	122.000	41.007	2.975	**0.041***	0.041
	Residuals	10.089.333	4	2.522.333													
	variant	44.083.333	1	44.083.333	6.275	0.087					O_MR	O_POT	191.667	76.516	2.505	0.087	0.087
	Residuals	21.076.667	3	7.025.556													
	variant	150.416.667	1	150.416.667	4.270	0.108					P_MR	P_POT	316.667	153.239	2.066	0.108	0.108
	Residuals	140.893.333	4	35.223.333													
	variant	1.232.667	1	1.232.667	1.880	0.242					B_POT	SUB	28.667	20.907	1.371	0.242	0.242
	Residuals	2.622.667	4	655.667													
	variant	12.241.200	1	12.241.200	4.164	0.134					O_POT	SUB	101.000	49.497	2.041	0.134	0.134
	Residuals	8.820.000	3	2.940.000													
	variant	16.667	1	16.667	0.035	0.860					P_POT	SUB	-3.333	17.714	-0.188	0.860	0.860
	Residuals	1.882.667	4	470.667													
	variant	47.051.556	2	23.525.778	0.875	0.464					B_MR	O_MR	-142.000	133.907	-1.060	0.570	0.989
	Residuals	161.380.000	6	26.896.667								P_MR	-162.667	133.907	-1.215	0.488	0.810
											O_MR	P_MR	-20.667	133.907	-0.154	0.987	1.000
	variant	196.683.556	2	98.341.778	19.988	0.002					B_BS	O_BS	-362.000	57.271	-6.321	**0.002***	0.002
	Residuals	29.520.000	6	4.920.000								P_BS	-188.667	57.271	-3.294	**0.038***	0.050
											O_BS	P_BS	173.333	57.271	3.027	0.053	0.070
Simpson dominance index	variant	9.923e -7	1	9.923e -7	0.384	0.569					B_MR	B_BS	8.133e -4	0.001	0.620	0.569	0.569
	Residuals	1.034e -5	4	2.585e -6													
							variant	1.190	1	0.275	O_MR	O_ BS	0.005	0.004	1.154	0.313	0.313
							variant	0.429	1	0.513	P_MR	P_BS	-0.006	0.007	-0.899	0.419	0.419
	variant	6.787e -5	1	6.787e -5	17.007	0.015					B_MR	B_POT	-0.007	0.002	-4.124	**0.015***	0.015
	Residuals	1.596e -5	4	3.991e -6													
	variant	6.694e -5	1	6.694e -5	8.910	0.058					O_MR	O_POT	-0.007	0.003	-2.985	0.058	0.058
	Residuals	2.254e -5	3	7.514e -6													
	variant	3.099e -6	1	3.099e -6	0.041	0.850					P_MR	P_POT	-0.001	0.007	-0.202	0.850	0.850
	Residuals	3.042e -4	4	7.605e -5													
	variant	1.012e -4	1	1.012e -4	32.639	0.005					B_POT	SUB	-0.008	0.001	-5.713	**0.005***	0.005
	Residuals	1.240e -5	4	3.100e -6													
	variant	2.543e -4	1	2.543e -4	34.126	0.010					O_POT	SUB	-0.015	0.002	-5.842	**0.010***	0.010
	Residuals	2.235e -5	3	7.451e -6													
	variant	2.445e -4	1	2.445e -4	47.712	0.002					P_POT	SUB	-0.013	0.002	-6.907	0.002	0.002
	Residuals	2.050e -5	4	5.124e -6													
	variant	1.119e -4	2	5.596e -5	1.113	0.388					B_MR	O_MR	0.007	0.006	1.224	0.483	0.800
	Residuals	3.016e -4	6	5.026e -5								P_MR	-7.360e -4	0.006	-0.127	0.991	1.000
											O_MR	P_MR	-0.008	0.006	-1.351	0.421	0.676

	variant	6.078e -5	2	3.039e -5	1.860	0.235					B_BS	O_BS	0.003	0.003	0.994	0.607	1.000
	Residuals	9.804e -5	6	1.634e -5								P_BS	0.006	0.003	1.928	0.211	0.306
											O_BS	P_BS	0.003	0.003	0.934	0.641	1.000
Gini-Simpson index	variant	1.042e -6	1	1.042e -6	0.392	0.565					B_MR	B_BS	-8.333e -4	0.001	-0.626	0.565	0.565
	Residuals	1.063e -5	4	2.657e -6													
							variant	1.190	1	0.275	O_MR	O_BS	-0.005	0.004	-1.151	0.314	0.314
							variant	0.429	1	0.513	P_MR	P_BS	0.006	0.007	0.900	0.419	0.419
	variant	6.801e -5	1	6.801e -5	16.785	0.015					B_MR	B_POT	0.007	0.002	4.097	**0.015***	0.015
	Residuals	1.621e -5	4	4.052e -6													
	variant	6.660e -5	1	6.660e -5	9.023	0.057					O_MR	O_POT	0.007	0.002	3.004	0.057	0.057
	Residuals	2.214e -5	3	7.382e -6													
	variant	3.082e -6	1	3.082e -6	0.040	0.850					P_MR	P_POT	0.001	0.007	0.201	0.850	0.850
	Residuals	3.044e -4	4	7.610e -5													
	variant	1.009e -4	1	1.009e -4	32.694	0.005					B_POT	SUB	0.008	0.001	5.718	**0.005***	0.005
	Residuals	1.234e -5	4	3.085e -6													
	variant	2.540e -4	1	2.540e -4	34.666	0.010					O_POT	SUB	0.015	0.002	5.888	**0.010***	0.010
	Residuals	2.199e -5	3	7.328e -6													
	variant	2.432e -4	1	2.432e -4	48.869	0.002					P_POT	SUB	0.013	0.002	6.991	**0.002***	0.002
	Residuals	1.991e -5	4	4.977e -6													
	variant	1.119e -4	2	5.594e -5	1.110	0.389					B_MR	O_MR	-0.007	0.006	-1.219	0.485	0.805
	Residuals	3.023e -4	6	5.038e -5								P_MR	7.667e -4	0.006	0.132	0.990	1.000
											O_MR	P_MR	0.008	0.006	1.352	0.421	0.676
	variant	6.083e -5	2	3.041e -5	1.860	0.235					B_BS	O_BS	-0.003	0.003	-0.999	0.604	1.000
	Residuals	9.813e -5	6	1.635e -5								P_BS	-0.006	0.003	-1.928	0.211	0.306
											O_BS	P_BS	-0.003	0.003	-0.929	0.644	1.000
Shannon diversity index	variant	0.013	1	0.013	0.587	0.486					B_MR	B_BS	-0.092	0.120	-0.766	0.486	0.486
	Residuals	0.086	4	0.021													
	variant	0.005	1	0.005	0.172	0.700					O_MR	O_ BS	0.056	0.134	0.415	0.700	0.700
	Residuals	0.108	4	0.027													
	variant	0.044	1	0.044	0.206	0.673					P_MR	P_BS	0.170	0.375	0.454	0.673	0.673
	Residuals	0.844	4	0.211													
	variant	0.156	1	0.156	8.061	0.047					B_MR	B_POT	0.322	0.113	2.839	**0.047***	0.047
	Residuals	0.077	4	0.019													
	variant	0.464	1	0.464	11.031	0.045					O_MR	O_POT	0.622	0.187	3.321	**0.045***	0.045
	Residuals	0.126	3	0.042													
							variant	1.190	1	0.275	P_MR	P_POT	0.613	0.376	1.629	0.179	0.179
	variant	0.040	1	0.040	5.853	0.073					B_POT	SUB	0.164	0.068	2.419	0.073	0.073
	Residuals	0.027	4	0.007													
	variant	0.235	1	0.235	6.840	0.079					O_POT	SUB	0.442	0.169	2.615	0.079	0.079
	Residuals	0.103	3	0.034													
	variant	0.054	1	0.054	6.629	0.062					P_POT	SUB	0.189	0.073	2.575	0.062	0.062
	Residuals	0.032	4	0.008													
	variant	0.503	2	0.252	1.644	0.270					B_MR	O_MR	-0.578	0.319	-1.810	0.245	0.361
	Residuals	0.918	6	0.153								P_MR	-0.316	0.319	-0.989	0.610	1.000
											O_MR	P_MR	0.262	0.319	0.821	0.705	1.000
	variant	0.882	2	0.441	22.065	0.002					B_BS	O_BS	-0.726	0.115	-6.285	**0.002***	0.002
	Residuals	0.120	6	0.020								P_BS	-0.578	0.115	-5.006	**0.006***	0.007
											O_BS	P_BS	0.148	0.115	1.279	0.456	0.744
	variant	1.261e -4	1	1.261e -4	2.254	0.208					B_MR	B_BS	-0.009	0.006	-1.501	0.208	0.208

	Residuals	2.239e -4	4	5.597e -5													
	variant	0.003	1	0.003	2.475	0.191					O_MR	O_ BS	0.041	0.026	1.573	0.191	0.191
	Residuals	0.004	4	0.001													
							variant	1.190	1	0.275	P_MR	P_BS	-0.035	0.032	-1.089	0.337	0.337
	variant	5.022e -4	1	5.022e -4	7.596	0.051					B_MR	B_POT	-0.018	0.007	-2.756	0.051	0.051
	Residuals	2.644e -4	4	6.611e -5													
	variant	6.446e -4	1	6.446e -4	7.516	0.071					O_MR	O_POT	-0.023	0.008	-2.742	0.071	0.071
	Residuals	2.573e -4	3	8.576e -5													
	variant	7.457e -4	1	7.457e -4	0.483	0.525					P_MR	P_POT	0.022	0.032	0.695	0.525	0.525
	Residuals	0.006	4	0.002													
Berger-Parker index	variant	0.002	1	0.002	43.058	0.003					B_POT	SUB	-0.041	0.006	-6.562	**0.003***	0.003
	Residuals	2.310e -4	4	5.775e -5													
	variant	0.005	1	0.005	68.884	0.004					O_POT	SUB	-0.061	0.007	-8.300	**0.004***	0.004
	Residuals	1.969e -4	3	6.563e -5													
	variant	0.006	1	0.006	79.542	< .001					P_POT	SUB	-0.066	0.007	-8.919	**< .001***	< .001
	Residuals	3.246e -4	4	8.115e -5													
							variant	3.289	2	0.193	B_MR	O_MR	0.026	0.027	0.956	0.628	1.000
												P_MR	-0.016	0.027	-0.588	0.831	1.000
											O_MR	P_MR	-0.041	0.027	-1.544	0.338	0.521
							variant	2.489	2	0.288	B_BS	O_BS	-0.025	0.021	-1.151	0.521	0.881
												P_BS	0.010	0.021	0.479	0.883	1.000
											O_BS	P_BS	0.035	0.021	1.630	0.305	0.463

B birch, O oak, P pine; MR field plant mycorrhizosphere, BS bulk soil, POT pot plant mycorrhizosphere, SUB control pot substrate, grey cells: not determined, asterisks: significant p values (< 0.05).

Table S4. One-Way ANOVA, Kruskal-Wallis test, post hoc test output for significance of differences between variants of the experiment in diversity indices of fungal communities.

Diversity Index	ANOVA						Kruskal-Wallis				Post Hoc comparisons						
	Cases	Sum of Squares	df	Mean Square	F	p	Factor	Statistic	df	p	Variant of comparisons		Mean Difference	SE	t	p tukey	p bonf
Richness	variant	2.090.667	1	2.090.667	1.052	0.363					B_MR	B_BS	37.333	36.397	1.026	0.363	0.363
	Residuals	7.948.667	4	1.987.167													
	variant	160.167	1	160.167	0.074	0.799					O_MR	O_BS	10.333	38.058	0.272	0.799	0.799
	Residuals	8.690.667	4	2.172.667													
	variant	9.204.167	1	9.204.167	3.155	0.150					P_MR	P_BS	78.333	44.100	1.776	0.150	0.150
	Residuals	11.668.667	4	2.917.167													
	variant	48.167	1	48.167	0.038	0.856					B_MR	B_POT	5.667	29.242	0.194	0.856	0.856
	Residuals	5.130.667	4	1.282.667													
	variant	326.700	1	326.700	0.085	0.789					O_MR	O_POT	16.500	56.516	0.292	0.789	0.789
	Residuals	11.498.500	3	3.832.833													
	variant	2.480.667	1	2.480.667	6.831	0.059					P_MR	P_POT	40.667	15.560	2.614	0.059	0.059
	Residuals	1.452.667	4	363.167													
	variant	8.893.500	1	8.893.500	40.425	0.003					B_POT	SUB	-77.000	12.111	-6.358	**0.003***	0.003
	Residuals	880.000	4	220.000													
							variant	0.333	1	0.564	O_POT	SUB	-64.500	57.088	-1.130	0.341	0.341
	variant	6.936.000	1	6.936.000	31.527	0.005					P_POT	SUB	-68.000	12.111	-5.615	**0.005***	0.005
	Residuals	880.000	4	220.000													
	variant	2.907.556	2	1.453.778	1.345	0.329					B_MR	O_MR	-23.333	26.844	-0.869	0.677	1.000

	Residuals	6.485.333	6	1.080.889								P_MR	-44.000	26.844	-1.639	0.301	0.457
											O_MR	P_MR	-20.667	26.844	-0.770	0.734	1.000
	variant	15.100.222	2	7.550.111	2.076	0.206					B_BS	O_BS	3.667	49.242	0.074	0.997	1.000
	Residuals	21.822.667	6	3.637.111								P_BS	-85.000	49.242	-1.726	0.271	0.405
											O_BS	P_BS	-88.667	49.242	-1.801	0.248	0.366
Simpson dominance index	variant	3.481e -4	1	3.481e -4	0.080	0.791					B_MR	B_BS	-0.015	0.054	-0.283	0.791	0.791
	Residuals	0.017	4	0.004													
	variant	0.005	1	0.005	0.663	0.461					O_MR	O_BS	0.060	0.073	0.814	0.461	0.461
	Residuals	0.032	4	0.008													
	variant	0.009	1	0.009	1.970	0.233					P_MR	P_BS	-0.078	0.056	-1.403	0.233	0.233
	Residuals	0.019	4	0.005													
	variant	1.236e -5	1	1.236e -5	3.606e -4	0.986					B_MR	B_POT	0.003	0.151	0.019	0.986	0.986
	Residuals	0.137	4	0.034													
							variant	0.333	1	0.564	O_MR	O_POT	-0.164	0.159	-1.032	0.378	0.378
	variant	0.002	1	0.002	0.447	0.540					P_MR	P_POT	0.033	0.050	0.668	0.540	0.540
	Residuals	0.015	4	0.004													
							variant	3.857	1	0.050	B_POT	SUB	0.203	0.147	1.382	0.239	0.239
							variant	3.000	1	0.083	O_POT	SUB	0.270	0.155	1.739	0.180	0.180
	variant	0.014	1	0.014	30.670	0.005					P_POT	SUB	0.096	0.017	5.538	**0.005***	0.005
	Residuals	0.002	4	4.474e -4													
	variant	0.016	2	0.008	1.913	0.228					B_MR	O_MR	0.100	0.053	1.869	0.227	0.333
	Residuals	0.026	6	0.004								P_MR	0.077	0.053	1.434	0.383	0.605
											O_MR	P_MR	-0.023	0.053	-0.435	0.903	1.000
	variant	0.033	2	0.017	2.338	0.177					B_BS	O_BS	0.025	0.069	0.359	0.932	1.000
	Residuals	0.043	6	0.007								P_BS	0.140	0.069	2.027	0.187	0.267
											O_BS	P_BS	0.115	0.069	1.667	0.291	0.440
Gini-Simpson index	variant	3.481e -4	1	3.481e -4	0.080	0.791					B_MR	B_BS	0.015	0.054	0.283	0.791	0.791
	Residuals	0.017	4	0.004													
	variant	0.005	1	0.005	0.662	0.461					O_MR	O_BS	-0.060	0.073	-0.814	0.461	0.461
	Residuals	0.032	4	0.008													
	variant	0.009	1	0.009	1.971	0.233					P_MR	P_BS	0.078	0.056	1.404	0.233	0.233
	Residuals	0.019	4	0.005													
	variant	1.233e -5	1	1.233e -5	3.598e -4	0.986					B_MR	B_POT	-0.003	0.151	-0.019	0.986	0.986
	Residuals	0.137	4	0.034													
							variant	0.333	1	0.564	O_MR	O_POT	0.164	0.159	1.032	0.378	0.378
	variant	0.002	1	0.002	0.447	0.540					P_MR	P_POT	-0.033	0.050	-0.669	0.540	0.540
	Residuals	0.015	4	0.004													
							variant	3.857	1	0.050	B_POT	SUB	-0.203	0.147	-1.382	0.239	0.239
							variant	3.000	1	0.083	O_POT	SUB	-0.270	0.155	-1.739	0.180	0.180
	variant	0.014	1	0.014	30.665	0.005					P_POT	SUB	-0.096	0.017	-5.538	**0.005***	0.005
	Residuals	0.002	4	4.474e -4													
	variant	0.016	2	0.008	1.913	0.228					B_MR	O_MR	-0.100	0.053	-1.869	0.227	0.332
	Residuals	0.026	6	0.004								P_MR	-0.077	0.053	-1.434	0.383	0.605
											O_MR	P_MR	0.023	0.053	0.435	0.903	1.000
	variant	0.033	2	0.017	2.338	0.178					B_BS	O_BS	-0.025	0.069	-0.359	0.932	1.000
	Residuals	0.043	6	0.007								P_BS	-0.140	0.069	-2.026	0.187	0.267
											O_BS	P_BS	-0.115	0.069	-1.667	0.292	0.440
	variant	0.162	1	0.162	0.971	0.380					B_MR	B_BS	0.329	0.334	0.985	0.380	0.380

	Residuals	0.669	4	0.167													
	variant	0.019	1	0.019	0.125	0.741					O_MR	O_BS	-0.114	0.321	-0.354	0.741	0.741
	Residuals	0.619	4	0.155													
	variant	0.922	1	0.922	3.879	0.120					P_MR	P_BS	0.784	0.398	1.970	0.120	0.120
	Residuals	0.951	4	0.238													
	variant	0.398	1	0.398	0.782	0.427					B_MR	B_POT	-0.515	0.583	-0.884	0.427	0.427
	Residuals	2.038	4	0.510													
	variant	0.124	1	0.124	0.187	0.694					O_MR	O_POT	0.322	0.743	0.433	0.694	0.694
	Residuals	1.988	3	0.663													
	variant	0.073	1	0.073	1.101	0.353					P_MR	P_POT	-0.221	0.210	-1.049	0.353	0.353
Shannon diversity index	Residuals	0.265	4	0.066													
	variant	2.940	1	2.940	7.521	0.052					B_POT	SUB	-1.400	0.511	-2.742	0.052	0.052
	Residuals	1.564	4	0.391													
							variant	3.000	1	0.083	O_POT	SUB	-1.568	0.690	-2.272	0.108	0.108
	variant	1.628	1	1.628	177.835	< .001					P_POT	SUB	-1.042	0.078	-13.34	**< .001***	< .001
	Residuals	0.037	4	0.009													
	variant	0.874	2	0.437	2.623	0.152					B_MR	O_MR	-0.669	0.333	-2.006	0.191	0.275
	Residuals	1.000	6	0.167								P_MR	-0.653	0.333	-1.959	0.203	0.293
											O_MR	P_MR	0.016	0.333	0.047	0.999	1.000
	variant	2.057	2	1.028	4.979	0.053					B_BS	O_BS	-0.226	0.371	-0.609	0.821	1.000
	Residuals	1.239	6	0.207								P_BS	-1.108	0.371	-2.986	0.055	0.073
											O_BS	P_BS	-0.882	0.371	-2.377	0.119	0.165
Berger-Parker index	variant	0.002	1	0.002	0.212	0.669					B_MR	B_BS	0.032	0.070	0.460	0.669	0.669
	Residuals	0.030	4	0.007													
	variant	0.012	1	0.012	0.795	0.423					O_MR	O_BS	0.090	0.101	0.891	0.423	0.423
	Residuals	0.061	4	0.015													
	variant	0.021	1	0.021	1.122	0.349					P_MR	P_BS	-0.118	0.111	-1.059	0.349	0.349
	Residuals	0.074	4	0.018													
	variant	7.639e -4	1	7.639e -4	0.013	0.914					B_MR	B_POT	0.023	0.195	0.116	0.914	0.914
	Residuals	0.228	4	0.057													
							variant	0.333	1	0.564	O_MR	O_POT	-0.185	0.180	-1.028	0.380	0.380
	variant	0.004	1	0.004	0.283	0.623					P_MR	P_POT	0.054	0.101	0.532	0.623	0.623
	Residuals	0.061	4	0.015													
							variant	0.429	1	0.513	B_POT	SUB	0.245	0.188	1.300	0.263	0.263
							variant	3.000	1	0.083	O_POT	SUB	0.365	0.174	2.100	0.127	0.127
	variant	0.037	1	0.037	11.694	0.027					P_POT	SUB	0.158	0.046	3.420	**0.027***	0.027
	Residuals	0.013	4	0.003													
	variant	0.012	2	0.006	0.496	0.632					B_MR	O_MR	0.088	0.089	0.983	0.613	1.000
	Residuals	0.072	6	0.012								P_MR	0.056	0.089	0.630	0.810	1.000
											O_MR	P_MR	-0.032	0.089	-0.353	0.934	1.000
	variant	0.075	2	0.037	2.401	0.171					B_BS	O_BS	0.030	0.102	0.297	0.953	1.000
	Residuals	0.093	6	0.016								P_BS	0.206	0.102	2.029	0.186	0.266
											O_BS	P_BS	0.176	0.102	1.732	0.269	0.402

B birch, O oak, P pine; MR field plant mycorrhizosphere, BS bulk soil, POT pot plant mycorrhizosphere, SUB control pot substrate, grey cells: not determined, asterisks: significant p values (< 0.05).

Ascomycota

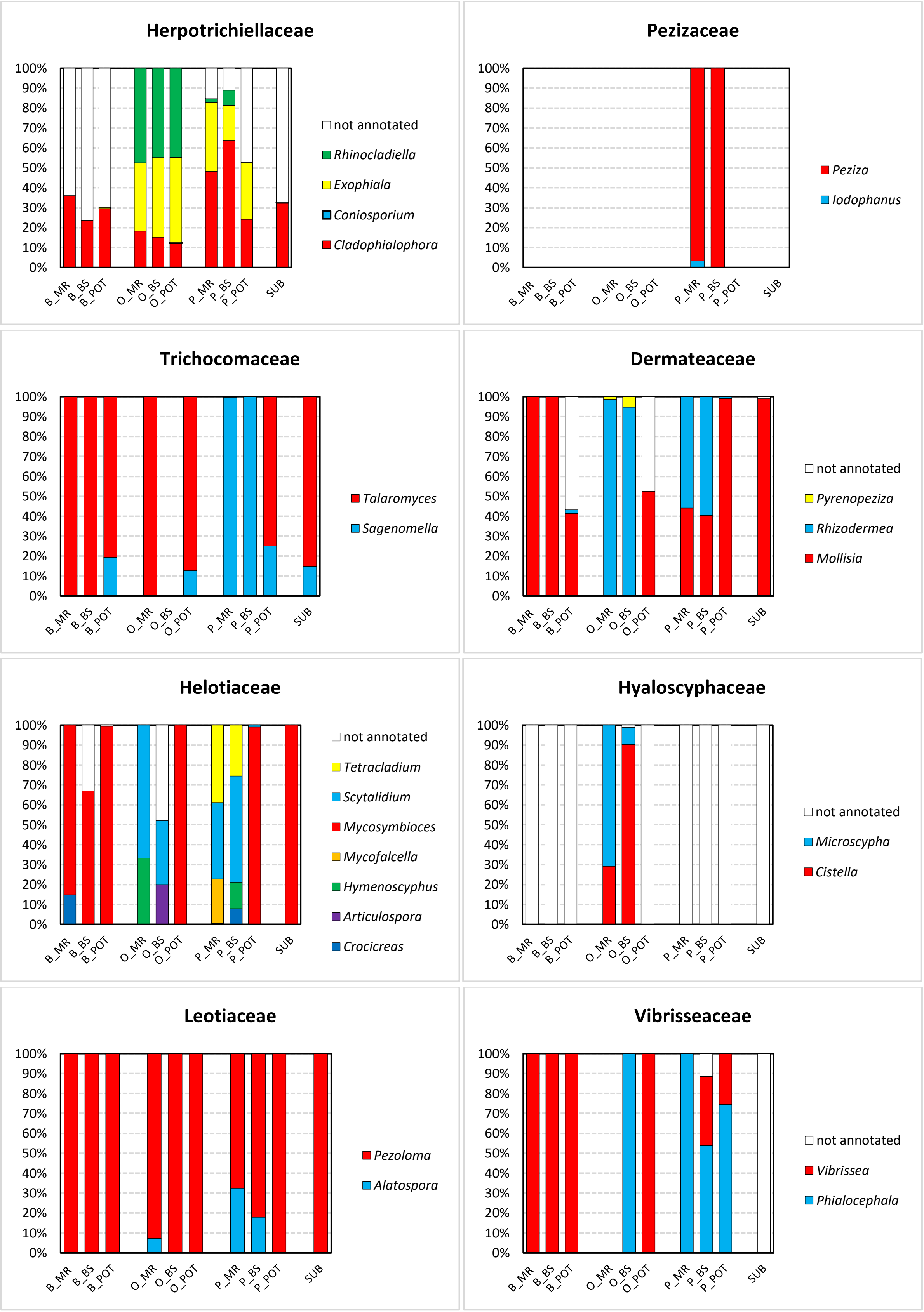
Herpotrichiellaceae
not annotated
Rhinocladiella
Exophiala
Coniosporium
Cladophialophora
Pezizaceae
Peziza
Iodophanus
Trichocomaceae
Talaromyces
Sagenomella
Dermateaceae
not annotated
Pyrenopeziza
Rhizodermea
Mollisia
Helotiaceae
not annotated
Tetracladium
Scytalidium
Mycosymbioces
Mycofalcella
Hymenoscyphus
Articulospora
Crocicreas
Hyaloscyphaceae
not annotated
Microscypha
Cistella
Leotiaceae
Pezoloma
Alatospora
Vibrisseaceae
not annotated
Vibrissea
Phialocephala
0%
10%
20%
30%
40%
50%
60%
70%
80%
90%
100%
B_MR
B_BS
B_POT
O_MR
O_BS
O_POT
P_MR
P_BS
P_POT
SUB

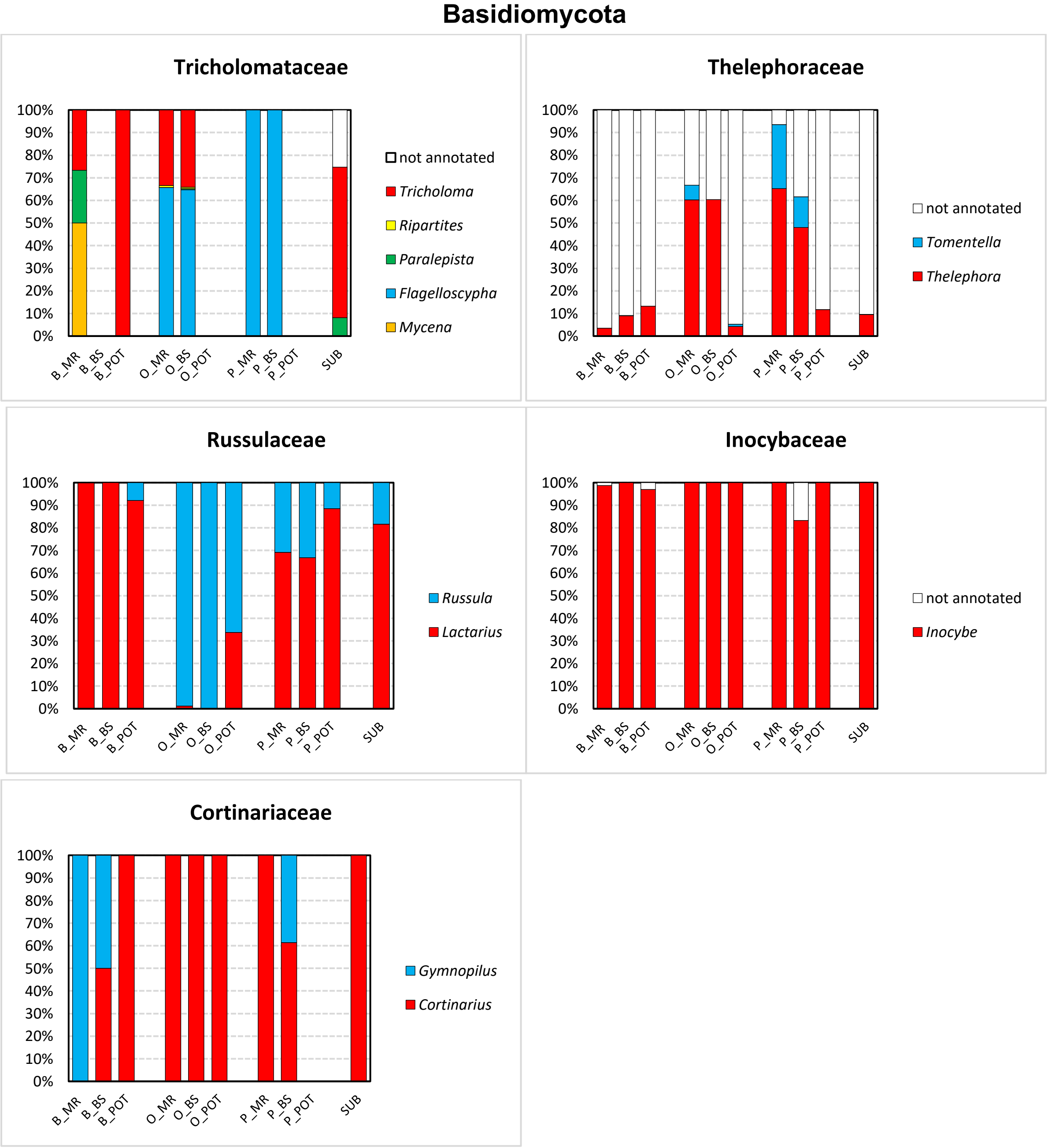

Figure S3. Characterization of the most representative fungal families at genus level. B, birch; O, oak; P, pine; MR, mycorrhizosphere of field plants; BS, bulk soil; POT, mycorrhizosphere of pot plants; SUB; control pot substrate. Some genera were the only members of their families and are therefore not shown with a diagram. So, for *Mortierellaceae*, 100 % *Mortierella* was present in each variant. *Serendipita* (100 % of *Serendipitaceae*) was present in the variants O_MR, O_BS and all pine variants, but absent from other variants. *Hebeloma* (the only *Hynemogastraceae* member) was observed in O_BS and O_POT as well as for all pine variants and absent from other variants. *Laccaria* (the only *Hydrangiaceae*) was present in all variants aside from O_MR and O_BS. *Tylospora* (*Atheliaceae*) was present only in P_MR and P_BS. *Penicillium* (*Aspergillaceae*), *Trichoderma* (*Hypocreaceae*) and *Pisolithus* (*Pisolithaceae*) were the only members of their families and present in all variants.

Table S5. Indices of similarity between fungal communities determined for trees within variants of the experiment.

Index	Estimate	s.e.	95%Lower	95%Upper
		B_MR		
Sorensen	0.202	0.013	0.177	0.227
Jaccard	0.078	0.006	0.066	0.090
Bray-Curtis	0.437	0.001	0.435	0.439
		O_MR		
Sorensen	0.199	0.007	0.187	0.212
Jaccard	0.077	0.003	0.071	0.082
Bray-Curtis	0.062	0.000	0.061	0.062
		P_MR		
Sorensen	0.145	0.009	0.128	0.162
Jaccard	0.054	0.004	0.047	0.061
Bray-Curtis	0.083	0.002	0.078	0.087
		B_BS		
Sorensen	0.267	0.007	0.253	0.281
Jaccard	0.108	0.004	0.101	0.115
Bray-Curtis	0.509	0.001	0.507	0.512
		O_BS		
Sorensen	0.180	0.011	0.159	0.201
Jaccard	0.068	0.005	0.059	0.077
Bray-Curtis	0.104	0.001	0.103	0.106
		P_BS		
Sorensen	0.149	0.005	0.140	0.158
Jaccard	0.055	0.002	0.052	0.059
Bray-Curtis	0.173	0.001	0.171	0.145
		B_POT		
Sorensen	0.199	0.007	0.186	0.213
Jaccard	0.077	0.003	0.071	0.083
Bray-Curtis	0.062	0.000	0.061	0.062
		O_POT		
Sorensen	0.124	0.002	0.120	0.128
Jaccard	0.066	0.001	0.064	0.068
Bray-Curtis	0.012	0.008	0.000	0.027
		P_POT		
Sorensen	0.225	0.005	0.216	0.235
Jaccard	0.088	0.002	0.084	0.093
Bray-Curtis	0.306	0.001	0.304	0.308
		SUB		
Sorensen	0.214	0.006	0.204	0.225
Jaccard	0.083	0.003	0.079	0.088
Bray-Curtis	0.118	0.001	0.116	0.119

B birch, O oak, P pine; MR field plant mycorrhizosphere, BS bulk soil, POT pot plant mycorrhizosphere, SUB control pot substrate.

Table S6. Highest abundant fungal families in different variants of the experiment.

Index	B_MR	B_BS	B_POT	O_MR	O_BS	O_POT	P_MR	P_BS	P_POT	SUB
Mortierellaceae	1	0	0	2	1	1	1	4	1	1
Thelephoraceae	24	6	44	4	3	37	11	15	31	1
Serendipitaceae	0	0	0	2	3	0	1	3	0	0
Russulaceae	7	15	0	47	53	0	7	11	0	0
Pisolithaceae	0	0	12	0	0	1	0	0	1	4
Atheliaceae	0	0	0	0	0	0	4	1	0	0
Tricholomataceae	0	0	0	3	3	0	0	0	0	0
Inocybaceae	34	42	5	0	0	5	29	18	19	1
Hymenogastraceae	0	0	0	0	0	0	4	0	0	0
Hydnangiaceae	0	0	6	0	0	0	0	0	0	0
Cortinariaceae	0	0	0	0	0	0	5	0	0	0
Hypocreaceae	0	0	2	1	2	1	5	1	3	2
Pezizaceae	0	0	0	0	0	0	2	1	0	0
Vibrisseaceae	0	0	0	0	0	2	1	1	2	1
Leotiaceae	19	8	13	7	2	12	6	4	14	23
Hyaloscyphaceae	8	12	4	0	1	10	0	0	8	9
Helotiaceae	0	2	0	0	2	0	0	0	0	1
Dermateaceae	0	0	3	0	0	2	1	1	3	7
Trichocomaceae	0	1	0	0	0	0	3	3	0	1
Aspergillaceae	0	0	1	1	0	0	1	1	1	3
Herpotrichiellaceae	1	3	2	22	13	15	6	4	4	10
less than 1%	1	2	3	3	5	1	3	10	3	8
unidentified	5	8	6	6	10	10	9	22	10	27

Five highest abundant families labeled in 100-80-60-40-20% black shadows; B, birch; O, oak; P, pine; MR, mycorrhizosphere of field plant; BS, bulk soil; POT, mycorrhizosphere of pot plant; SUB, control pot substrate.

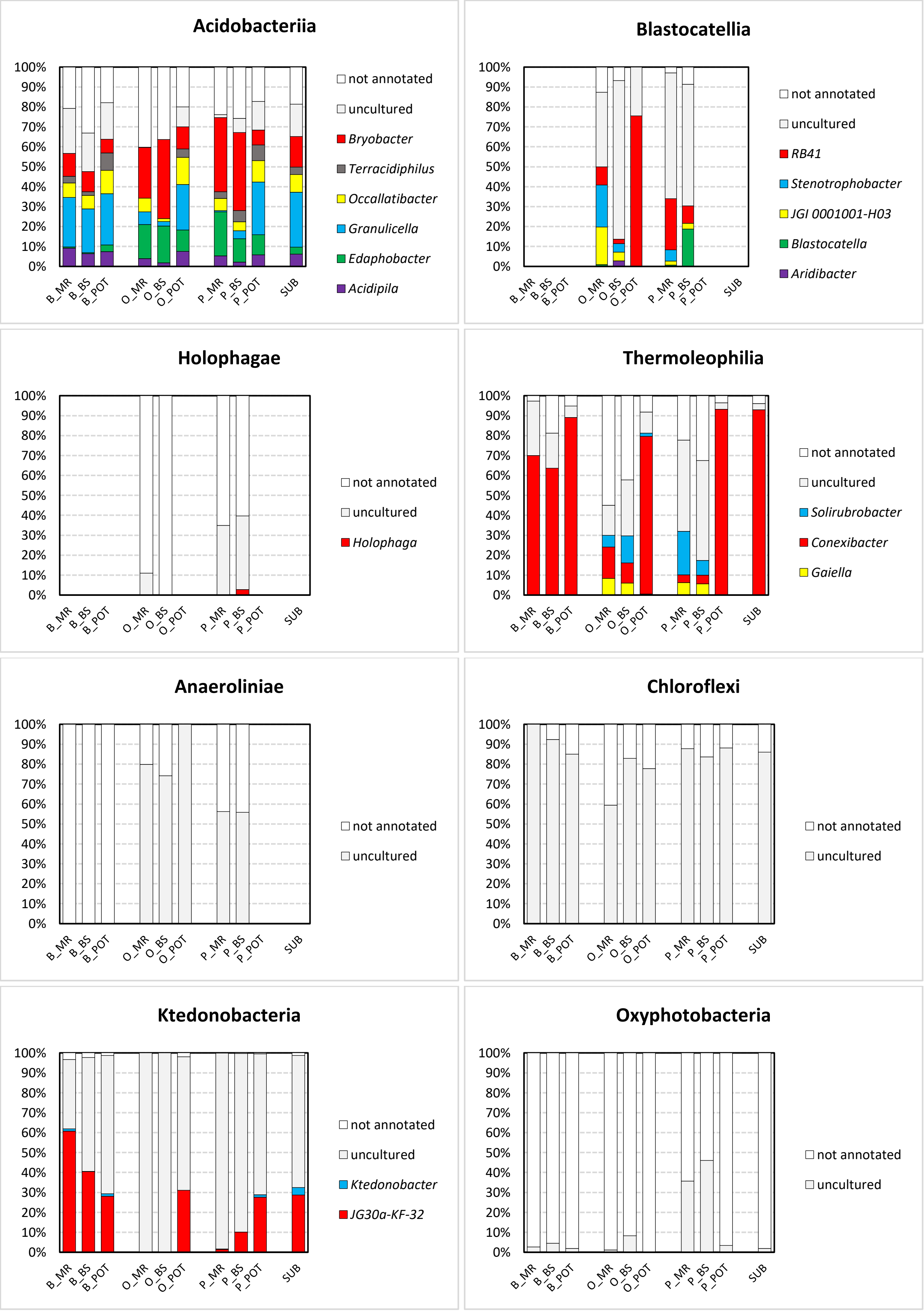
Acidobacteriia
not annotated
uncultured
Bryobacter
Terracidiphilus
Occallatibacter
Granulicella
Edaphobacter
Acidipila
Blastocatellia
not annotated
uncultured
RB41
Stenotrophobacter
JGI 0001001-H03
Blastocatella
Aridibacter
Holophagae
not annotated
uncultured
Holophaga
Thermoleophilia
not annotated
uncultured
Solirubrobacter
Conexibacter
Gaiella
Anaerolineae
not annotated
uncultured
Chloroflexi
not annotated
uncultured
Ktedonobacteria
not annotated
uncultured
Ktedonobacter
JG30a-KF-32
Oxyphotobacteria
not annotated
uncultured
0%
10%
20%
30%
40%
50%
60%
70%
80%
90%
100%
B_MR
B_BS
B_POT
O_MR
O_BS
O_POT
P_MR
P_BS
P_POT
SUB

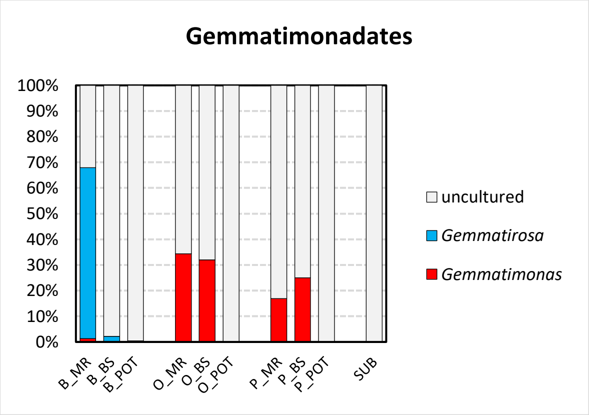
Gemmatimonadates
uncultured
Gemmatirosa
Gemmatimonas
B_MR
B_BS
B_POT
O_MR
O_BS
O_POT
P_MR
P_BS
P_POT
SUB

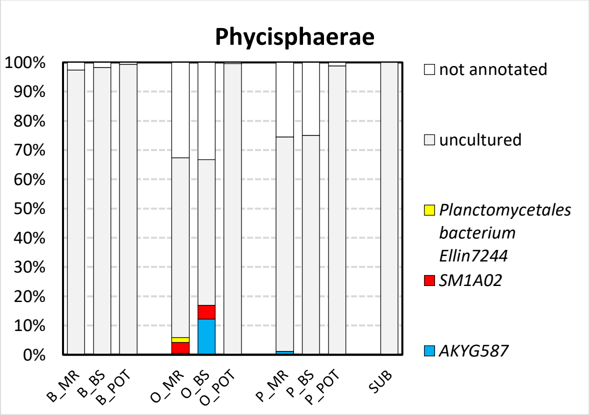
Phycisphaerae
not annotated
uncultured
Planctomycetales bacterium Ellin7244
SM1A02
AKYG587
B_MR
B_BS
B_POT
O_MR
O_BS
O_POT
P_MR
P_BS
P_POT
SUB

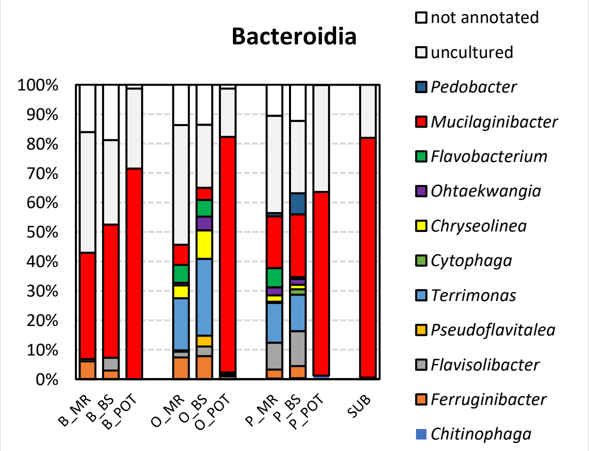
Bacteroidia
not annotated
uncultured
Pedobacter
Mucilaginibacter
Flavobacterium
Ohtaekwangia
Chryseolinea
Cytophaga
Terrimonas
Pseudoflavitalea
Flavisolibacter
Ferruginibacter
Chitinophaga
B_MR
B_BS
B_POT
O_MR
O_BS
O_POT
P_MR
P_BS
P_POT
SUB

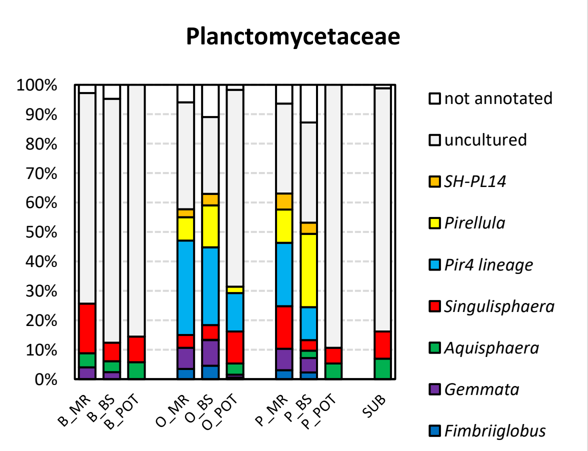
Planctomycetaceae
not annotated
uncultured
SH-PL14
Pirellula
Pir4 lineage
Singulisphaera
Aquisphaera
Gemmata
Fimbriiglobus
B_MR
B_BS
B_POT
O_MR
O_BS
O_POT
P_MR
P_BS
P_POT
SUB

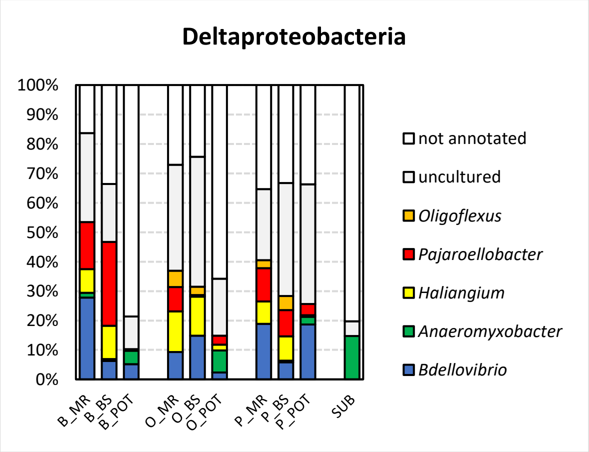
Deltaproteobacteria
not annotated
uncultured
Oligoflexus
Pajaroellobacter
Haliangium
Anaeromyxobacter
Bdellovibrio
B_MR
B_BS
B_POT
O_MR
O_BS
O_POT
P_MR
P_BS
P_POT
SUB

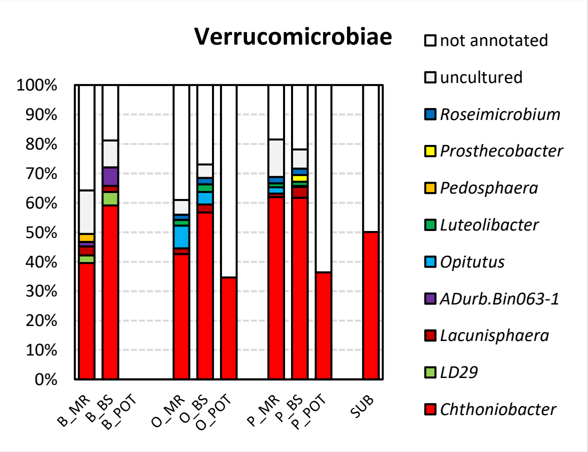
Verrucomicrobiae
not annotated
uncultured
Roseimicrobium
Prosthecobacter
Pedosphaera
Luteolibacter
Opitutus
ADurb.Bin063-1
Lacunisphaera
LD29
Chthoniobacter
B_MR
B_BS
B_POT
O_MR
O_BS
O_POT
P_MR
P_BS
P_POT
SUB

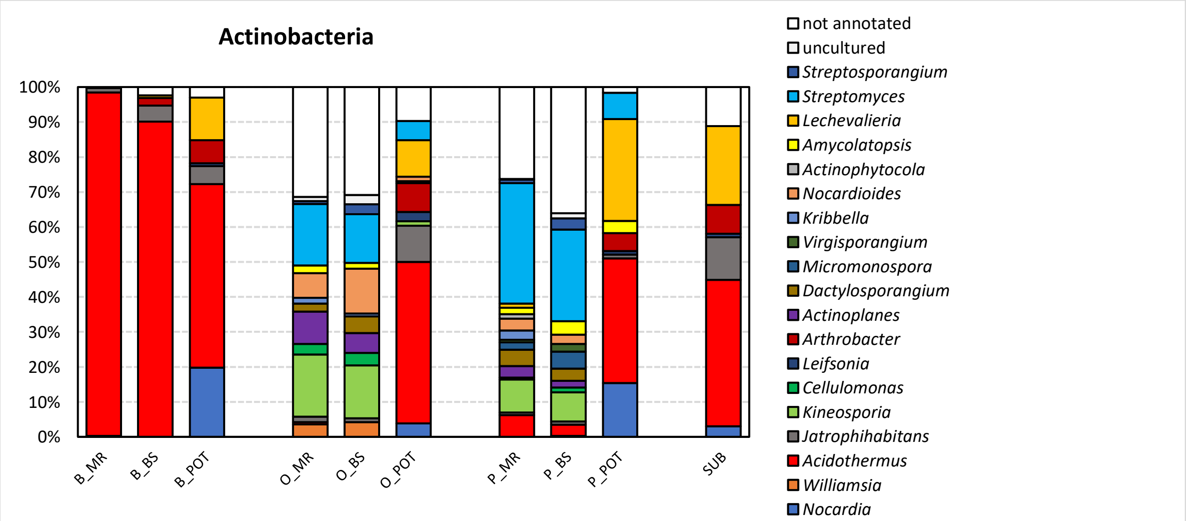
Actinobacteria
not annotated
uncultured
Streptosporangium
Streptomyces
Lechevalieria
Amycolatopsis
Actinophytocola
Nocardioides
Kribbella
Virgisporangium
Micromonospora
Dactylosporangium
Actinoplanes
Arthrobacter
Leifsonia
Cellulomonas
Kineosporia
Jatrophihabitans
Acidothermus
Williamsia
Nocardia
B_MR
B_BS
B_POT
O_MR
O_BS
O_POT
P_MR
P_BS
P_POT
SUB

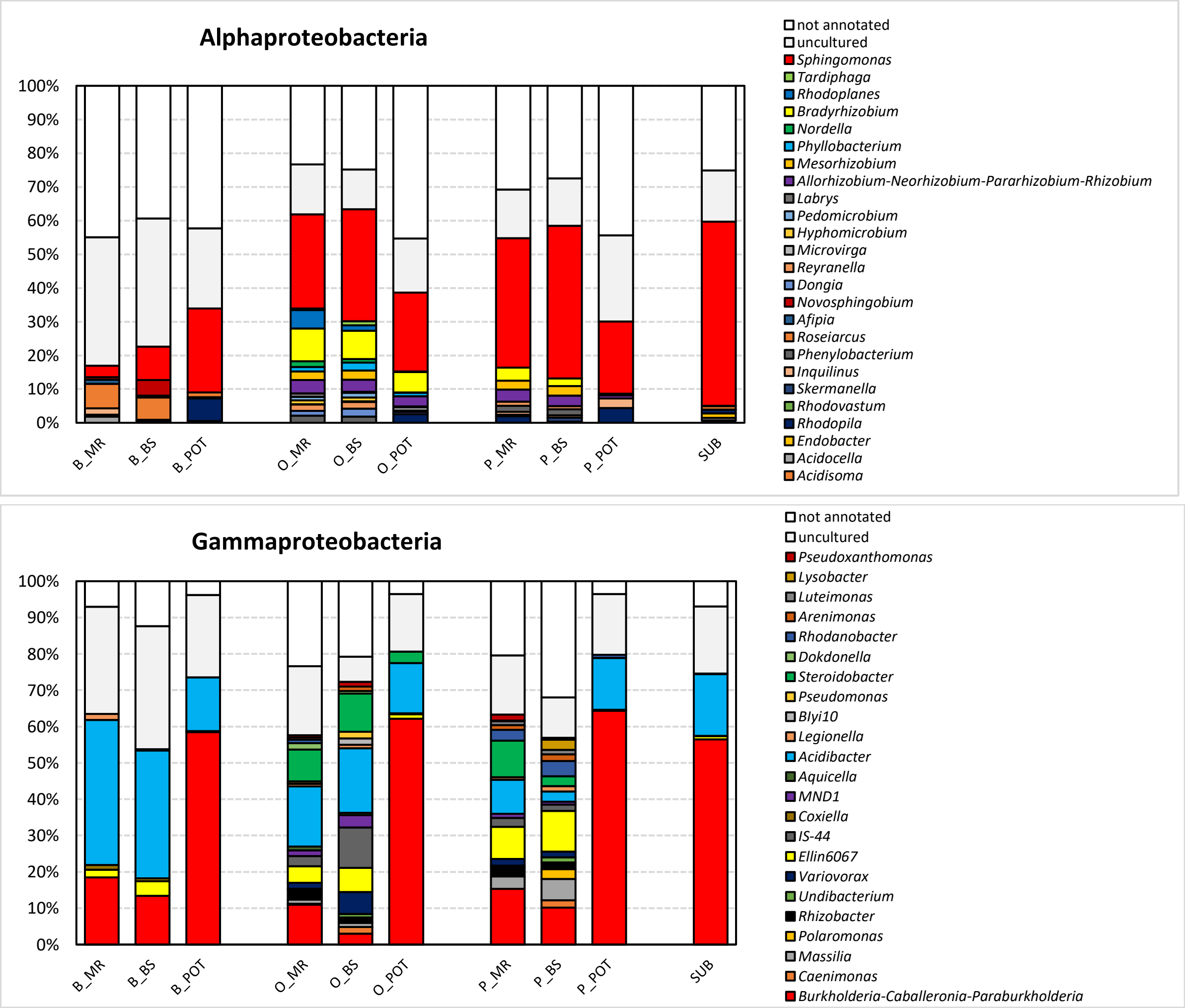

Figure S4. Characterization of the most representative bacterial classes at genus level. B, birch; O, oak; P, pine; MR, mycorrhizosphere of field plants; BS, bulk soil; POT, mycorrhizosphere of pot plants; SUB, control pot substrate; y-axis represents relative abundance of bacterial genera within a considered bacterial class.

Table S7. Highest abundant bacterial classes in different variants of the experiment.

Index	B_MR	B_BS	B_POT	O_MR	O_BS	O_POT	P_MR	P_BS	P_POT	SUB
Verrucomicrobiae	4	2	0	10	8	0	6	4	0	0
Gammaproteobacteria	6	5	4	10	10	5	10	10	7	2
Deltaproteobacteria	1	2	0	2	3	0	2	2	1	0
Alphaproteobacteria	20	13	16	28	26	21	25	28	17	15
Planctomycetacia	4	5	1	4	4	2	3	2	1	1
Phycisphaerae	9	7	12	4	3	10	4	3	14	7
Gemmatimonadetes	0	2	0	2	1	0	1	2	0	0
Oxyphotobacteria	3	1	5	1	1	4	1	2	3	3
Ktedonobacteria	11	16	20	0	0	19	5	4	20	26
Chloroflexia	0	1	0	0	0	1	1	1	0	1
Anaerolineae	0	0	0	1	1	0	2	2	0	0
Bacteroidia	8	10	3	13	12	4	12	16	6	1
Thermoleophilia	0	1	2	2	2	2	1	1	1	6
Actinobacteria	4	3	5	4	3	7	8	5	5	5
Acidimicrobiia	0	0	1	0	0	1	1	0	1	3
Holophagae	0	0	0	0	0	0	2	2	0	0
Blastocatellia (Gr. 4)	0	0	0	4	9	0	2	2	0	0
Acidobacteriia	20	20	17	4	2	13	5	4	16	11
less than 1%	2	1	0	1	1	0	1	1	0	0
unidentified	6	10	13	8	13	11	10	9	8	18

Five highest abundant families labeled in 100-80-60-40-20% black shadows; B, birch; O, oak; P, pine; MR, mycorrhizosphere of field plant; BS, bulk soil; POT, mycorrhizosphere of pot plant; SUB, control pot substrate.

Table S8. Indices of similarity between bacterial communities determined for trees within variants of the experiment.

Index	Estimate	s.e.	95%Lower	95%Upper
B_MR				
Sorensen	0.572	0.002	0.569	0.575
Jaccard	0.308	0.001	0.305	0.311
Bray-Curtis	0.508	0.001	0.505	0.511
O_MR				
Sorensen	0.317	0.002	0.314	0.321
Jaccard	0.134	0.001	0.132	0.136
Bray-Curtis	0.265	0.001	0.262	0.268
P_MR				
Sorensen	0.222	0.002	0.218	0.226
Jaccard	0.087	0.001	0.085	0.089
Bray-Curtis	0.144	0.001	0.142	0.146
B_BS				
Sorensen	0.541	0.002	0.537	0.546
Jaccard	0.282	0.002	0.2785	0.286
Bray-Curtis	0.520	0.002	0.518	0.523
O_BS				
Sorensen	0.413	0.001	0.411	0.415
Jaccard	0.190	0.001	0.189	0.191
Bray-Curtis	0.351	0.001	0.348	0.353
P_BS				
Sorensen	0.242	0.001	0.240	0.244
Jaccard	0.096	0.001	0.095	0.097
Bray-Curtis	0.199	0.001	0.197	0.202
B_POT				
Sorensen	0.756	0.002	0.753	0.760
Jaccard	0.509	0.003	0.504	0.514
Bray-Curtis	0.784	0.001	0.781	0.786
O_POT				
Sorensen	0.535	0.001	0.533	0.538
Jaccard	0.365	0.001	0.363	0.368
Bray-Curtis	0.614	0.002	0.609	0.619
P_POT				
Sorensen	0.673	0.002	0.669	0.678
Jaccard	0.407	0.002	0.403	0.412
Bray-Curtis	0.708	0.002	0.704	0.712
SUB				
Sorensen	0.729	0.003	0.723	0.735
Jaccard	0.473	0.004	0.465	0.480
Bray-Curtis	0.814	0.002	0.811	0.817

B birch, O oak, P pine; MR field plant mycorrhizosphere, BS bulk soil, POT pot plant mycorrhizosphere, SUB control pot substrate.

Table S9. R-statistic of pairwise ANOSIM of field sampling sites based on the relative abundance of the most representative fungal taxa.

	Field Birches sampling sites	Field Oaks sampling sites	Field Pines sampling sites
Field Birches sampling sites			
Field Oaks sampling sites	**1.00***		
Field Pines sampling sites	**0.45***	**0.79***	

Asterisks represent significant differences ($p < 0.05$).

Table S10. PERMANOVA p-values of pairwise comparisons between field sampling sites based on the relative abundance of the most representative fungal taxa.

	Field Birches sampling sites	Field Oaks sampling sites	Field Pines sampling sites
Field Birches sampling sites			
Field Oaks sampling sites	**0.01***		
Field Pines sampling sites	**0.02***	**0.01***	

Asterisks represent significant differences ($p < 0.05$).

Table S11. SIMPER analysis demonstrating the contribution (%) of the most abundant fungal families to Bray-Curtis dissimilarity between field sampling sites.

	Field Birches sampling sites	Field Oaks sampling sites	Field Pines sampling sites
Field Birches sampling sites			
Field Oaks sampling sites	Russulaceae (27.5%)<br>Inocybaceae (26.4%)<br>Herpotrichiellaceae (10.6%)		
Field Pines sampling sites	Inocybaceae (19.9%)<br>Thelephoraceae (13.1%)<br>Russulaceae (12.7%)	Russulaceae (31.8%)<br>Inocybaceae (17.7%)<br>Herpotrichiellaceae (9.9%)	

Only fungal families with a contribution higher than 10% are included in the table.

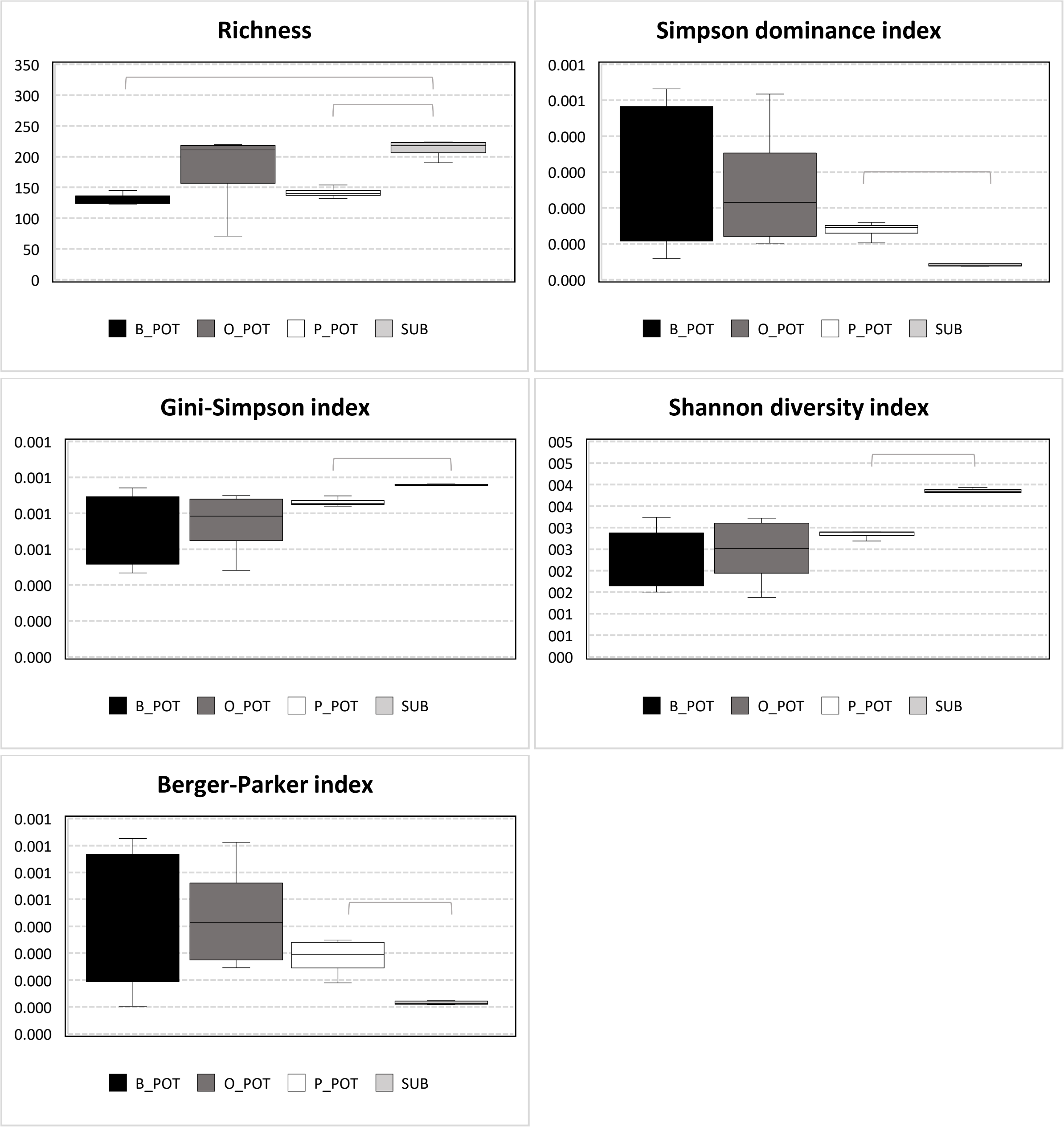

Figure S5. Pairwise comparisons of fungal community diversity indices based on ASVs between pot plant mycorrhizosphere and control pot substrate. B, birch; O, oak; P, pine; POT, mycorrhizosphere of pot plant; SUB, control pot substrate. Brackets indicate significant differences ($p < 0.05$).

Table S12. ANOVA test output of pairwise comparisons between field plant mycorrhizosphere, corresponding pot plant mycorrhizosphere and control pot substrate based on the relative abundance of the most representative fungal taxa.

Mean rank within groups	41.47
Mean rank between groups	101.5
R	0.6319
P	0.0001

Table S13. R-statistics of pairwise ANOSIM between field plant mycorrhizosphere, corresponding pot plant mycorrhizosphere and control pot substrate based on the relative abundance of the most representative fungal taxa.

	B_MR	B_POT	O_MR	O_POT	P_MR	P_POT	SUB
B_POT	0.63						
O_POT			0.75				
P_POT					0.30		
SUB	1	1	1	0.5	0.59		

B birch, O oak, P pine; MR field plant mycorrhizosphere, BS bulk soil, POT pot plant mycorrhizosphere, SUB control pot substrate. Asterisks represent significant values ($p < 0.05$).

Table S14. PERMANOVA test output of pairwise comparisons between field plant mycorrhizosphere, corresponding pot plant mycorrhizosphere and control pot substrate based on the relative abundance of the most representative fungal taxa.

Total sum of squares	4.236
Within-group sum of squares	1.385
F	4.46
P	0.0001

Table S15. PERMANOVA p-values of pairwise comparisons field plant mycorrhizosphere, pot plant mycorrhizosphere and control pot substrate based on the relative abundance of the most representative fungal taxa.

	B_MR	B_POT	O_MR	O_POT	P_MR	P_POT	SUB
B_POT	0.10						
O_POT			0.11				
P_POT					0.20		
SUB	0.10	0.10	0.10	0.10	0.10		

B birch, O oak, P pine; MR field plant mycorrhizosphere, BS bulk soil, POT pot plant mycorrhizosphere, SUB control pot substrate. Asterisks represent significant values ($p < 0.05$).

Table S16. R-statistics of pairwise ANOSIM of field sampling sites based on the relative abundance of the most representative bacterial taxa.

	Field Birches sampling site	Field Oaks sampling site	Field Pines sampling site
Field Birches sampling site			
Field Oaks sampling site	**0.99***		
Field Pines sampling site	**0.76***	0.23	

Asterisks represent significant differences ($p < 0.05$).

Table S17. PERMANOVA p-values of pairwise comparisons between field sampling sites based on the relative abundance of the most representative bacterial taxa.

	Field Birches sampling site	Field Oaks sampling site	Field Pines sampling site
Field Birches sampling site			
Field Oaks sampling site	**0.01***		
Field Pines sampling site	**0.01***	0.22	

Asterisks represent significant differences ($p < 0.05$).

Table S18. SIMPER analysis demonstrates the contribution (%) of the most abundant bacterial classes to Bray-Curtis dissimilarity between field sampling sites.

	Field Birches sampling site	Field Oaks sampling site	Field Pines sampling site
Field birches sampling site			
Field Oaks sampling site	Acidobacteriia (21.85%)<br>Ktedonobacteria (17.15%)<br>Alphaproteobacteria (13.89%)		
Field Pines sampling site	Acidobacteriia (22.04%)<br>Alphaproteobacteria (14.87%)<br>Ktedonobacteria (14.46%)	Alphaproteobacteria (12.2%)<br>Verrucomicrobiae (11.27%)<br>Blastocatellia (11.25%)	

Only bacterial classes with a contribution higher than 10% are included in the table.

Table S19. ANOSIM test output of pairwise comparisons between field plant mycorrhizosphere, pot plant mycorrhizosphere and control pot substrate based on the relative abundance of the most representative bacterial taxa.

Mean rank within groups	34.68
Mean rank between groups	102.3
R	0.7113
P	0.0001

Table S20. R-statistics of pairwise ANOSIM between field plant mycorrhizosphere, corresponding pot plant mycorrhizosphere and control pot substrate based on the relative abundance of the most representative bacterial taxa.

	B_MR	B_POT	O_MR	O_POT	P_MR	P_POT	SUB
B_POT	0.56						
O_POT			1				
P_POT					0.70		
SUB	1	1	1	1	0.81	1	

B birch, O oak, P pine; MR field plant mycorrhizosphere, BS bulk soil, POT pot plant mycorrhizosphere, SUB control pot substrate. Asterisks represent significant values ($p < 0.05$).

Table S21. PERMANOVA test output of pairwise comparisons between field plant mycorrhizosphere, pot plant mycorrhizosphere and control pot substrate based on the relative abundance of the most representative bacterial taxa.

Total sum of squares	1.474
Within-group sum of squares	0.2391
F	11.19
P	0.0001

Table S22. PERMANOVA p-values of pairwise comparisons field plant mycorrhizosphere, pot plant mycorrhizosphere and control pot substrate based on the relative abundance of the most representative bacterial taxa.

	B_MR	B_POT	O_MR	O_POT	P_MR	P_POT	SUB
B_POT	0.10						
O_POT			0.10				
P_POT					0.10		
SUB	0.10	0.10	0.10	0.10	0.10	0.10	

B birch, O oak, P pine; MR field plant mycorrhizosphere, BS bulk soil, POT pot plant mycorrhizosphere, SUB control pot substrate. Asterisks represent significant values ($p < 0.05$).
